# Uncovering nonlinear patterns in time-sensitive prehospital breathing emergencies: an exploratory machine learning study

**DOI:** 10.1186/s12911-025-03046-z

**Published:** 2025-06-03

**Authors:** Peter Hill, Daniel Jonsson, Jakob Lederman, Peter Bolin, Veronica Vicente

**Affiliations:** 1 Department for Specialized Care, The Health and Medical Care Administration, Stockholm, Sweden; 2 The Ambulance Medical Service in Stockholm (AISAB), Stockholm, Sweden; 3 Academic EMS in Stockholm, Stockholm, Sweden; 4 Department of Clinical Science and Education, Karolinska Institutet, Södersjukhuset, Stockholm, SE-118 83 Sweden; 5 Department of Urban Planning and Environment, KTH Royal Institute of Technology, Stockholm, Sweden; 6 Department of Learning, Informatics, Management and Ethics (LIME), Karolinska Institutet, Stockholm, Sweden

**Keywords:** Emergency medical services, Response time, Machine learning, Time-Sensitive conditions, Breathing problems, Clinical decision support systems

## Abstract

**Background:**

Timely prehospital care is crucial for patients presenting with high-risk time-sensitive (HRTS) conditions. However, the interplay between response time and demographic factors in patients with breathing problems remains insufficiently understood. In this exploratory study, we applied machine learning (ML) to examine how emergency medical response time, age, and sex jointly influence the probability of encountering HRTS conditions.

**Methods:**

A retrospective observational analysis was conducted on 132,395 prehospital missions in Stockholm (2017–2022). Multiple ML models, random forest, gradient boosting, neural networks, and logistic regression were trained to probe potential nonlinear patterns and interactions, not with the primary goal of predictive accuracy. Model performance was evaluated using sensitivity, specificity, and area under the curve (AUC) measures. However, partial dependence (PD) and individual conditional expectation (ICE) plots were the principal tools illustrating how response time, age, and sex shape HRTS likelihood.

**Results:**

PD and ICE plots revealed that older age (> 60 years) was consistently associated with a higher probability of HRTS. Moreover, patients over 60 years displayed a complex, rising risk at prolonged response times exceeding two hours. Gradient boosting offered the best (though modest) classification metrics, with an AUC of 0.66 and an F1-score of 0.55. We emphasize that these metrics, while necessary for completeness, were secondary to our aim of characterizing nonlinear relationships.

**Conclusions:**

Our findings underscore the exploratory value of ML in identifying subtle relationships and interactions among response time, age, and sex for time-sensitive breathing emergencies. These results highlight opportunities to refine dispatch protocols, develop age- and sex-focused screening questions, and revisit lower-priority calls after extended wait times. Future work should incorporate richer data and refine these insights for potential predictive use.

**Clinical trial number:**

Not applicable.

**Supplementary Information:**

The online version contains supplementary material available at 10.1186/s12911-025-03046-z.

## Introduction

Prehospital emergency care encompasses both Emergency Medical Call Centres (EMCC) and Emergency Medical Services (EMS), forming an integrated system aimed at timely patient interventions. Response time, the interval between an EMCC call and EMS arrival, is critical in conditions such as cardiac arrest, stroke, and trauma. While many studies emphasize the importance of rapid response, others find limited or no association with improved outcomes [1–4].

In Sweden, EMCC personnel categorize calls into three priority levels: Priority 1 (immediately life-threatening), Priority 2 (time-sensitive but non-life-threatening), and Priority 3 (non-time-sensitive) [5]. However, EMCC assessments have been found to have varied levels of accuracy [6]. EMCCs face several inherent challenges when triaging emergency calls over the phone. First, language barriers and emotional distress, such as agitation or panic on the part of the caller, often interfere with the dispatcher’s ability to elicit clear, accurate information. Second, because calls are seldom placed by the actual patient, the information provided may be incomplete, indirect, or simply less reliable. As a result, there can be a mismatch between the urgency initially assigned by the call center and the actual clinical condition observed by emergency responders on arrival. These discrepancies in urgency assessment can lead to misjudgments about resource allocation and response time, ultimately influencing patient outcomes [6, 7]. Studies indicate that over-triage, where the EMCC assigns a higher urgency level than the actual clinical condition warrant, occurs in approximately 44% of cases. Conversely, under-triage, where the assigned urgency is lower than the patient’s true medical severity, occurs in about 20% of cases [6].

On-scene, EMS personnel use triage tools like The Rapid Emergency Triage and Treatment System (RETTS). RETTS is based on both vital signs and symptoms and chief complaints. In Sweden, RETTS categorizes patients into five color-coded priority levels, but in our study setting only four are used—Red, Orange, Yellow, and Green [8–10]. Because Red and Orange both signify heightened clinical urgency, we combine them into a single “High Risk of Time-Sensitive Condition” (HRTS) group. This combined categorization aligns with established practice in our EMS region and other reported uses of RETTS, where Red/Orange often represent the tier most likely to require immediate intervention [8, 9].

A growing body of literature applies machine learning (ML) to predict triage outcomes or priority levels in EMCC, with promising results. Recent work has developed models for dispatch triage [11] and utilized deep ensemble architectures [12] to enhance emergency medical dispatch decisions. Others have explored random forest–based call triage [13] natural language processing of call transcripts [14] and advanced algorithms to more quickly detect critical events such as out-of-hospital cardiac arrest [15].

Beyond algorithmic choices, feature engineering has also proven crucial for boosting predictive accuracy. Novel approaches, such as combining structured data with text-based clinical notes or geospatial information, have further refined ML-driven triage models. For example, integrating advanced feature engineering techniques can identify subtle interactions between demographic or call-specific factors, leading to better performance in patient triage [9] [16]. Recent work has highlighted the importance of incorporating both quantitative and qualitative data into ML-based pipelines, demonstrating improved results when these richer feature sets are employed [9] [16].

Collectively, these efforts underscore the potential of ML in improving prioritization, reducing misclassification, and streamlining EMS resource utilization. However, most published research focuses on predictive performance metrics (e.g., AUC, F1-score) as the principal measures of success. Like a recent study in this domain [17] we emphasize an exploratory approach: while we report standard performance measures, the principal objective is to probe how age, sex, and especially response time are associated with the likelihood of encountering HRTS condition among patients initially reported to have breathing problems.

Patients with breathing problems represent a high-risk group in prehospital care, as they often present with acute respiratory distress and experience higher mortality rates than other groups [18]. Moreover, recent work by Ruge et al. [19] involving more than 600,000 ED visits in Sweden found that age was independently associated with increased short-term mortality across all RETTS-A priority levels, suggesting that traditional triage protocols may overlook or under-triage older adults. A subsequent multi-center study by Malmer et al. [20] further confirmed that adding age as a factor in RETTS triage substantially improved short-term mortality predictions. These findings emphasize that advanced age, combined with diminished respiratory function [21] may particularly predispose older adults to time-sensitive complications if they do not receive accurate triage. Despite these factors, many existing ML-based triage tools either exclude or only minimally address how demographic and clinical complexities affect triage outcomes for respiratory complaints.

This study focuses on these patients due to their clinical importance, aiming to fill this gap in literature. Although many more recent studies now exist on EMS dispatch procedures and response intervals, earlier foundational work continues to inform present-day protocols and benchmarking.

Traditional triage protocols or single-rule systems may overlook nonlinear patterns, such as a sudden spike in risk at certain ages or specific intervals of delayed EMS response. Machine learning techniques excel at capturing these more complex interactions, providing a data-driven way to identify subtle risk factors and optimize dispatch decisions. By integrating EMCC priority designations, on-scene triage (RETTS), patient demographic details, and response times, ML-based methods can more accurately map which factors that could have an association to encountering an HRTS condition in breathing emergencies. This approach not only extends the existing literature on EMCC triage models but also addresses a critical gap: applying advanced predictive analytics to a patient group disproportionately having a time-sensitive respiratory condition.

In this study, we investigate how EMS response times, age, and sex jointly influence HRTS condition among patients with breathing problems. The potential for nonlinear interactions—where risk might spike or flatten at certain thresholds—makes ML particularly valuable for discovery. Our work seeks to use ML to explore these patterns rather than strictly to produce a clinical predictive tool. In doing so, we build on prior work in ML-based triage while responding to calls in the literature to examine more granular clinical contexts (e.g., respiratory emergencies) that have received comparatively less attention. Ultimately, our goal is to deepen understanding of time-sensitive prehospital care for respiratory complaints and to provide a scalable, data-driven model that could guide more precise dispatch and resource allocation decisions in EMCC settings.

### Objective

To examine the relationship between response time and the likelihood of HRTS (equivalent to Red/Orange in RETTS) in patients initially assessed with breathing problems and to understand how age and sex interact with response time in shaping these probabilities.

## Materials and methods

### Study design

The study was conducted as a retrospective observational analysis of over one million prehospital missions in Stockholm, Sweden, between 2017 and 2022. The study adhered to STROBE guidelines [22]. Data were obtained from Region Stockholm’s VAL databases [23] offering pseudonymized, individual-level data. Machine learning was employed to explore nonlinear relationships. Throughout, our use of ML was exploratory: the central goal was to detect and visualize potential nonlinearities and interactions. Traditional classification metrics were evaluated to gauge model discrimination but were not the primary outcomes. Specifically, gradient boosting, a method that builds many small decision trees and combines them, was used to assess how response time, age, and sex impact the probability of HRTS [24]. Variables such as age, sex, and response time were analysed because of their impact on patient outcome [2–4] [25–27].

### Participants

The initial dataset (*N* = 1,297,858) was refined based on inclusion and exclusion criteria (Table 1), resulting in a final subset of 132,395 observations. No missing data were present after preprocessing. Information was retrieved from Region Stockholm about skewed timestamps in a few cases, which would give negative time values and extreme time values. That was handled by excluding the cases with negative response times and response times exceeding 10 h.

**Table 1:** Inclusion and exclusion criteria and resulting sample sizes. Sequential filters reduced the original EMS call dataset (*N* = 1,297,858) to a final analytic sample of 132,395 breathing‑problem missions that Met all study criteria

Initial dataset *N* = 1 297 858 observations	
Exclusion criteria	Number of excluded observations
Dispatch priority not 1, 2, or 3 (Transport assignments)	*n* ** = 1 165 463**
EMS unit type not Emergency Ambulance	
Year not between 2017 and 2022	
Response time not between 0 and 10 h*	
Intrahospital transports	
Reason of contact to EMCC not “Breathing problems”	
**Observations included***n* = **132 395 observations**	

* Observations with skewed timestamps caused by technical issues in the prehospital digital platform FRAPP were excluded. Specifically, data with negative time values or response times exceeding 10 h were considered highly unlikely on the basis of the authors’ domain expertise and were therefore omitted from the analysis

### Variables and features

**Fig. 1:**
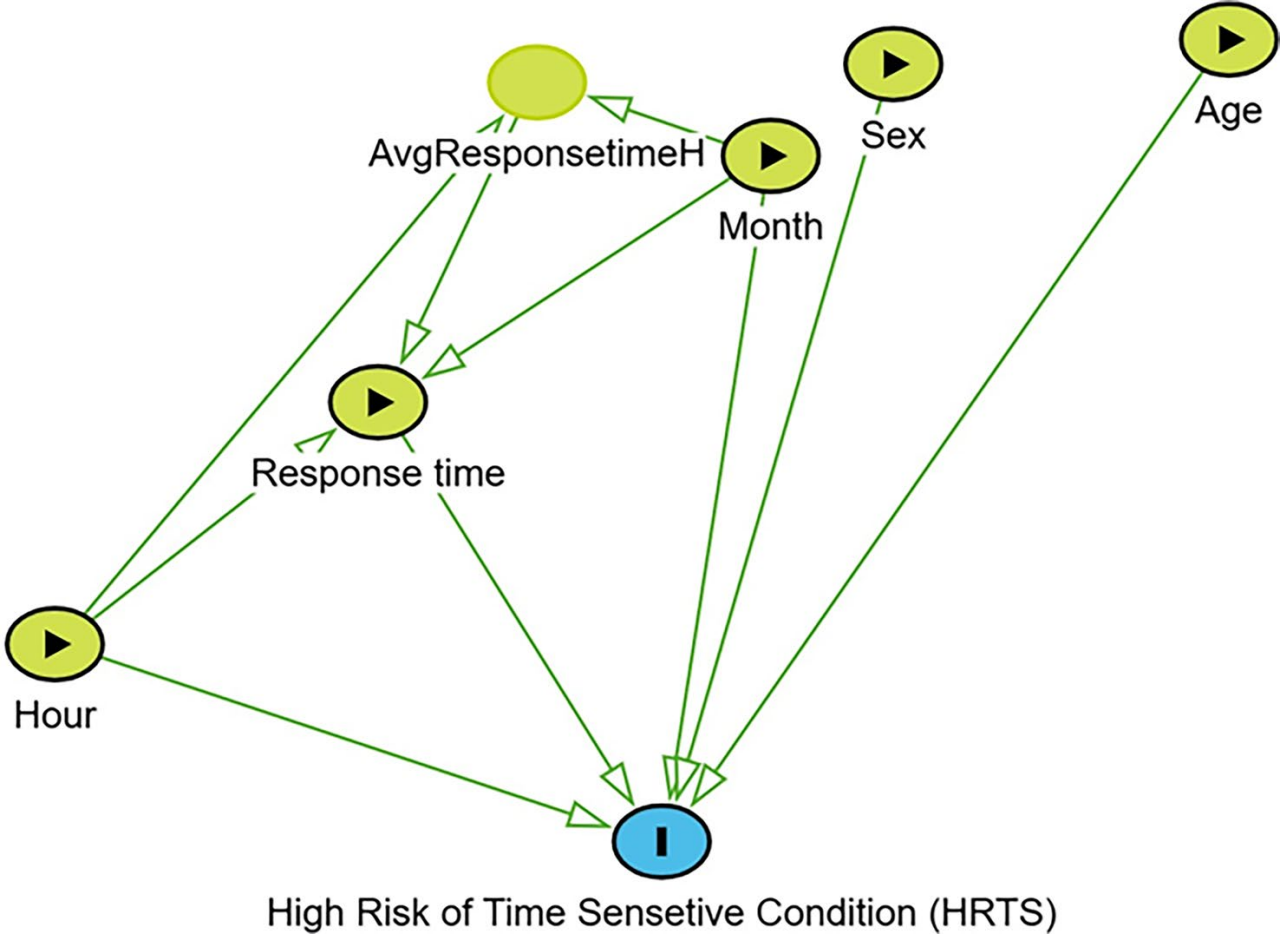
Directed acyclic graph illustrating the hypothesized relationships among the study variables age, sex, month, hour of day, response time, and average response time, and the outcome of high-risk, time-critical condition. Green arrows indicate the direction of presumed causal influence in the model [16]

A Directed Acyclic Graph (DAG) was constructed to guide which variables to include, ensuring alignment with our objective of linking response time to HRTS (Fig. 1) [16]. Key variables included age, response time, month, and hour of the day. Our outcome of interest was whether the patient had HRTS (equivalent to Red/Orange in RETTS). Since no hospital interventions or treatments were made to the patient, and RETTS is measured by the first assessment by EMS (it can be updated during EMS care process), measurement bias was minimized.

An overview of features is found in Appendix A. Detailed program code for feature engineering and machine learning methods is available in Appendix B.

### Statistical software

The study employed SAS Enterprise Guide 8.2 for data standardization and manipulation, ensuring adherence to rigorous statistical procedures. SAS Model Studio 8.5 was used for detailed data analysis, offering control over model settings and hyperparameter tuning. Code for these analyses is documented in Appendix B, ensuring transparency and reproducibility.

### Machine learning models

Machine learning models are suitable to describe the association between the variables in this study since the variables of interest have nonlinear effects on each other (Fig. 1), and the objective is to investigate a possible association between response time and HRTS.

The dataset was split into training (60%), validation (30%), and testing (10%) subsets. Models evaluated included Random Forest, Gradient Boosting, Neural Networks, Ensemble and Logistic Regression [24] [28–32]. We selected the ML approaches to encompass distinct operational logics and capture a diverse set of predictive behaviors (Table 2).

**Table 2:** Overview of the machine learning algorithms evaluated in the study, detailing each model’s conceptual basis, operational mechanics, and justification for inclusion in predicting time-sensitive conditions among patients with breathing problems

Model	Explanation	How it operates	Why it was included
**Random Forest**	An ensemble of decision trees, typically using a “bagging” approach (bootstrap aggregating). It reduces variance by combining multiple weak learners [33].	- Trains multiple trees on bootstrapped samples of the original dataset. - Each tree votes for a predicted class (classification) or outputs a value (regression). - The final prediction is based on majority vote or averaging [33].	Random Forest’s robustness, interpretability of feature importance, and proven track record in triage-oriented research made it a natural candidate to explore diverse aspects of emergency data [34].
**Gradient Boosting**	A boosting technique that builds a strong model by sequentially adding weak learners (often small decision trees), each one correcting the errors of the previous ensemble [29].	- Fits a new tree to the current “residuals” (the mistakes made by the ensemble so far). - Each tree’s contribution is weighted according to how well it reduces the overall error. - Repeats in multiple iterations to refine predictions [29].	Gradient Boosting can capture subtle, non-linear interactions among features and often yields high accuracy, making it especially suitable for complex healthcare data where multiple factors drive outcomes [35].
**Neural Networks**	Models inspired by biological neurons, consisting of interconnected layers (input, hidden, output). They excel at capturing highly complex or non-linear relationships [36].	- Passes input data through neuron-like layers that apply weights and nonlinear activation functions. - Learns by adjusting these weights to minimize a loss function via backpropagation. - May have multiple hidden layers to detect deeper patterns [36].	Neural Networks are included to explore their capability in recognizing complicated relationships among response times, age, and sex—potentially improving triage accuracy beyond what simpler models can detect.
**Ensemble Methods**	Any approach that combines multiple individual models to achieve better predictive performance than a single model. Random Forest and Gradient Boosting are specific examples [37].	- Blends outputs from different base models (e.g., averaging, voting, stacking). - Aims to offset weaknesses of any single model by leveraging the strengths of many. - Typically yields higher accuracy and robustness compared to stand-alone models [37].	Ensemble Methods (beyond Random Forest and Gradient Boosting) allow for further experimentation in combining different algorithms, potentially enhancing triage performance by balancing each algorithm’s biases.
**Logistic Regression**	A statistical technique for binary classification, despite the “regression” in its name. Uses a logistic (sigmoid) function to estimate the probability of a certain class [38].	- Applies a linear combination of features (weights and intercept). - Transforms that linear combination with the sigmoid function to get a probability between 0 and 1. - Ideal where relationships between features and the outcome are roughly linear [38].	Logistic Regression serves as a transparent, baseline model for comparing more advanced techniques, ensuring interpretability and offering a benchmark for predictive performance in emergency triage research.

**Fig. 2:**
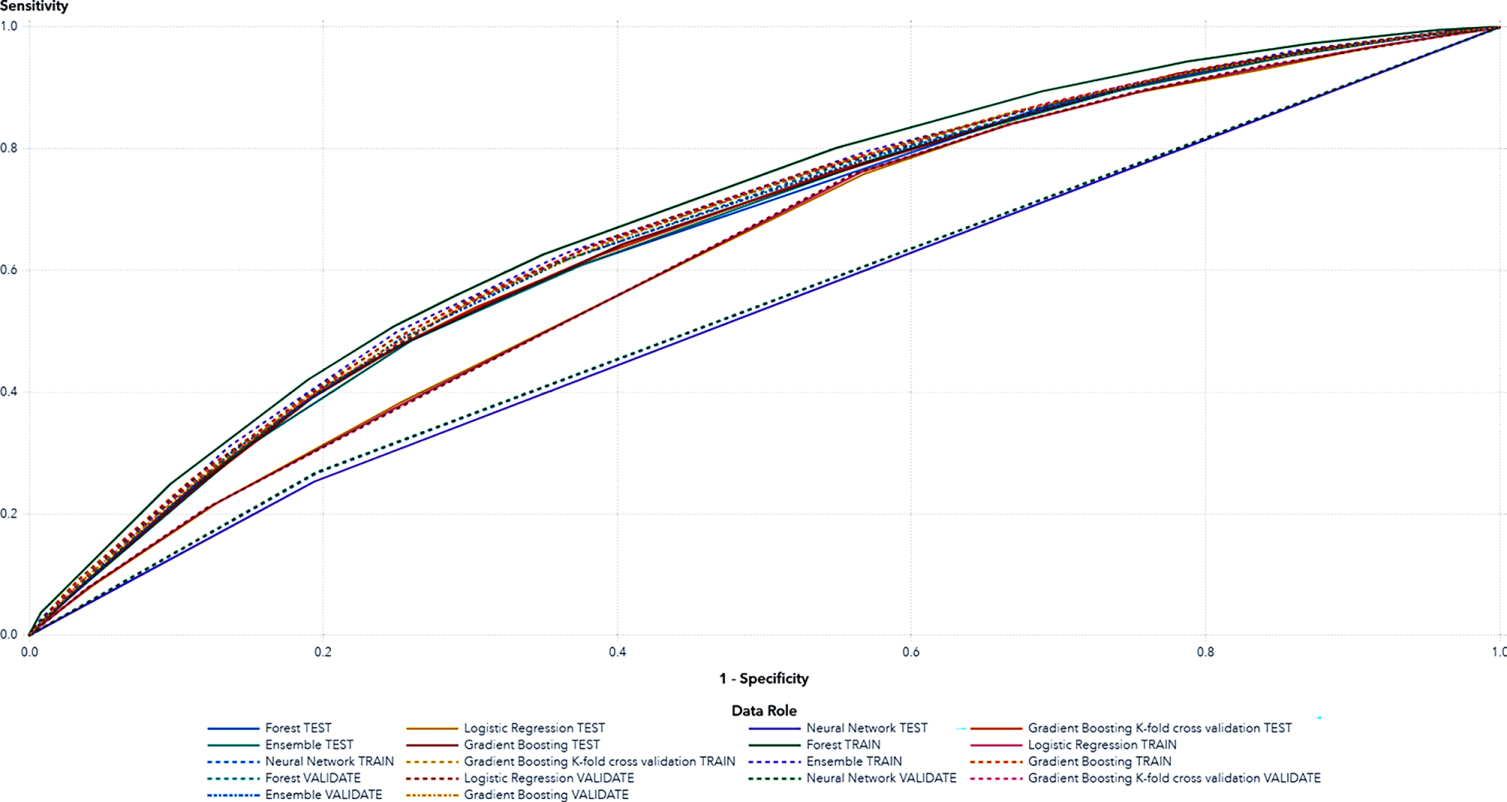
Receiver operating characteristic (ROC) curves comparing the performance of various machine learning models—Random Forest, Ensemble, Neural Network, Logistic Regression, and Gradient Boosting—across training, validation, and test datasets. Each curve illustrates the trade-off between sensitivity (y-axis) and 1-specificity (x-axis) at different classification thresholds. Gradient Boosting demonstrated the strongest overall performance, including under a K-fold cross-validation scheme, as evidenced by curves closer to the top-left corner

The choice was guided by two factors: first, some of these methods have been underexplored in prior triage-focused research, offering an opportunity to expand on existing knowledge; second, certain algorithms have demonstrated strong predictive performance in previous studies of EMS outcomes. A recent systematic review on ML in patient triage highlights these methods as especially well-suited for predicting triage outcomes, further supporting our selection [34]. Evaluating how well the model separates distributions, Gradient boosting outperformed random forest, neural networks, and logistic regression models achieving the highest Kolmogorov–Smirnov (KS) Index on the test data [39]. Although 10-fold cross-validation is often cited as a robust method for validating predictive models, we opted for a traditional split of the dataset into training, validation and testing partitions. The primary reason for this choice was our relatively large dataset (*n* = 132,395), which allowed us to maintain sufficiently large training, validation, and test subsets while ensuring that each partition captured representative variability in the data. Additionally, to evaluate the robustness of our modeling approach, we performed a 10-fold cross-validation procedure specifically on the Gradient Boosting Model. Its performance was almost identical to our training-validation-test approach; in fact, when comparing the KS Index measures, the split-sample method slightly outperformed the 10-fold cross-validation. Consequently, we decided to retain the training-validation-test scheme since it yielded marginally superior predictive performance while maintaining interpretability and computational efficiency (Table 3; Fig. 2). While we report performance measures, the real focus was uncovering how the probability of HRTS changes with response time, age, and sex, particularly via partial dependence (PD) and individual conditional expectation (ICE) plots.

**Table 3:** Model comparison showing performance metrics for each candidate algorithm used to predict high-risk, time-sensitive (HRTS) conditions in patients with breathing emergencies. gradient boosting emerged as the champion, with the highest Kolmogorov–Smirnov (KS) value (0.2397) and a misclassification rate of 0.3687. The table also includes a gradient boosting model trained with K-fold cross-validation, random forest (“forest”), neural network, an ensemble method, and logistic regression for completeness

Algorithm	Misclass. rate	Accuracy	Gini coeff.	Lift	AUC	F1 Score	KS Stat	False Pos. rate	False disc. rate	Captured resp. %
Gradient Boosting	0.3687	0.627	0.3192	1.4013	0.6596	0.5544	0.2397	0.3159	0.4401	7.01
Gradient Boosting K-fold	0.4201	0.63	0.3208	1.4048	0.6604	0.5521	0.2369	0.3039	0.4349	7.02
Forest	0.423	0.6271	0.3151	1.4334	0.6576	0.5492	0.2348	0.3073	0.4386	7.17
Ensemble	0.4218	0.6322	0.313	1.4013	0.6565	0.5271	0.2334	0.2599	0.4228	7.01
Logistic Regression	0.4674	0.5935	0.2401	1.3476	0.62	0.4423	0.1909	0.2512	0.4736	6.74
Neural Network	0.5068	0.5774	0.0593	1.1559	0.5297	0	0.0591	0		5.78

The autotuning feature in the software was used for guidance to an optimal hyperparameter configuration. In the champion model the learning rate is 0.34. A lower learning rate often yields better generalization, at the cost of needing more trees. The maximum tree levels were set to 2. Deeper trees can capture more complex patterns, but with the risk of overfitting. The subsampling rate of 0.8 was used to reduce variance and overfitting. The L1 and L2 penalties were fine tuned by trying different values while monitoring validation loss. The leaves were constraint to 4 to catch the true signal and reduce the risk of fit noise in the training data. Early stopping where set to 5 to minimize risk of fit the training noise rather than the generalizable patterns. The number of bins were 100 and gave appropriate splits and results (Table 4).

**Table 4:**
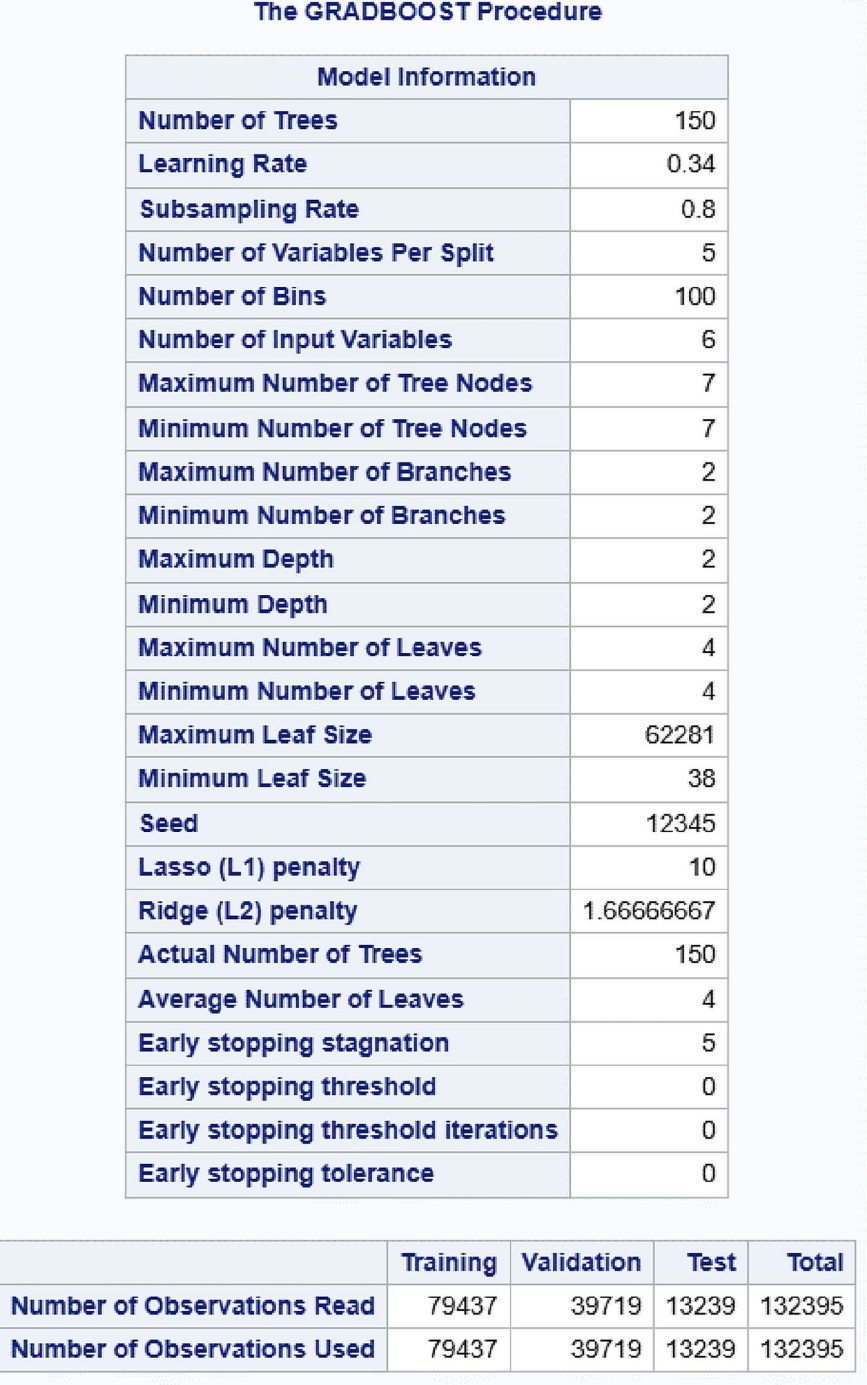
Final gradient boosting (GRADBOOST) model hyperparameters and data partition details. Shown are the chosen tree-related parameters (e.g., number of trees, learning rate, maximum leaves) along with the training (*n* = 79,437), validation (*n* = 39,719), and test (*n* = 13,239) subsets used from the total 132,395 observations

Since the objective is to investigate the association between variables that are nonlinear, partial dependence (PD) and Individual Conditional Expectation (ICE) plots were used to interpret results. PD plots and ICE plots are visualization tools used to interpret machine learning models by illustrating how input features influence predictions. PD plots provide a global view by showing the average effect of a single feature on the model’s predictions while holding other variables constant. In contrast, ICE plots extend this concept by displaying predictions for individual observations, making it possible to detect heterogeneous effects that may be masked in the averaged PD plot. While standard metrics can indicate model discrimination, they offer limited insight into why certain variables matter. PD and ICE plots reveal how predicted risk of HRTS changes with one variable (e.g., response time) when other variables are held constant. This is pivotal in an exploratory analysis aiming to identify possible causal or correlational structures in the data [34]. An example is a graph that shows how the predicted risk of HRTS changes as response time increases, for different ages or sexes. ICE plots show how each individual patient’s predicted risk responds to changes in one variable, revealing hidden patterns that might not be captured by average effects alone [40]. Traditional scatterplots where used to in-depth analyse the prediction for each observation in different groups.

### Ethical considerations

This study complied with ethical guidelines and received approval from the Swedish Ethical Review Authority (Dnr 2022-03701-01). The study adhered to the principles of the Declaration of Helsinki and good clinical practice standards [41, 42].

## Results

**Fig. 3:**
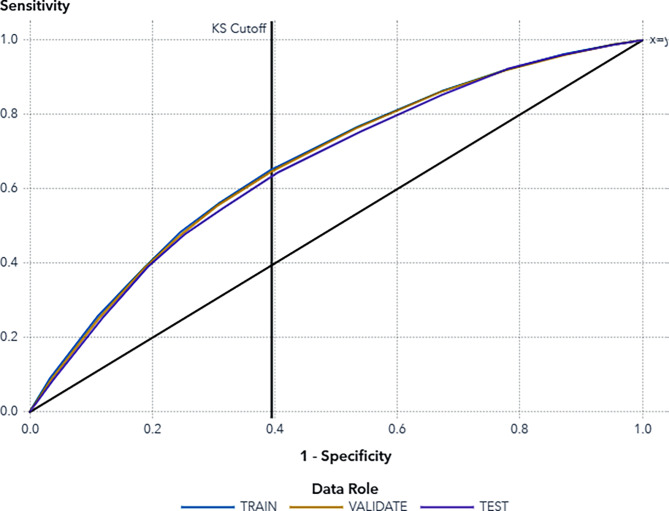
ROC curve for the used model. The KS cutoff reference line is drawn at the value of 1-specificity where the greatest difference between sensitivity and 1-specificity is observed for the Validate partition. The KS cutoff line is drawn at the cutoff value 0.45, where the 1-specificity value is 0.4046 and the sensitivity value is 0.6443 in the test data

Among the models evaluated, the Gradient Boosting model demonstrated the highest performance achieving a Youden’s Index (measure the balance between sensitivity and specificity) of 0.2397^39^, Area Under Curve (AUC) 0,6596 and a misclassification rate of 0.3730 in the test data with a sensitivity of the model of 63% and a specificity of the model of 61%. While not perfect, this score compares favorably to the accuracy of standard EMCC prioritization [6]. The classification rate is reasonable considering the relatively few variables on which the prediction is made. The Kolmogorov-Smirnov (KS) cutoff point was established at a threshold value of 0.45 (Fig. 3). We stress that these metrics reflect the difficulty of predicting HRTS solely from limited, non-clinical data and do not imply that the model is ready for clinical triage decisions. Our primary interest lay in how the model captured the influence of response time, age, and sex on HRTS.

**Fig. 4:**
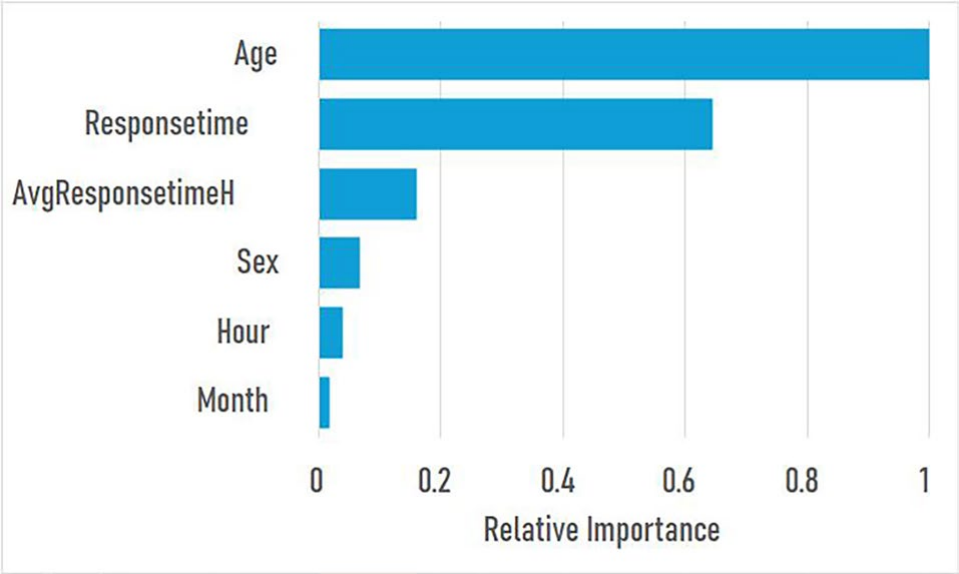
The plot shows the 6 most important features, as determined by the relative importance calculated using the Gradient Boosting model. The most important input for this model is Age, indicating that age highly influences the probability of HRTS. The input Response time has a relative importance of 0.64, for example, which means it is 0.64 times as important as Age

The model’s most important predictors were age, followed by response time in seconds, and average response time current hour of the emergency call in seconds (Fig. 4).

**Fig. 5:**
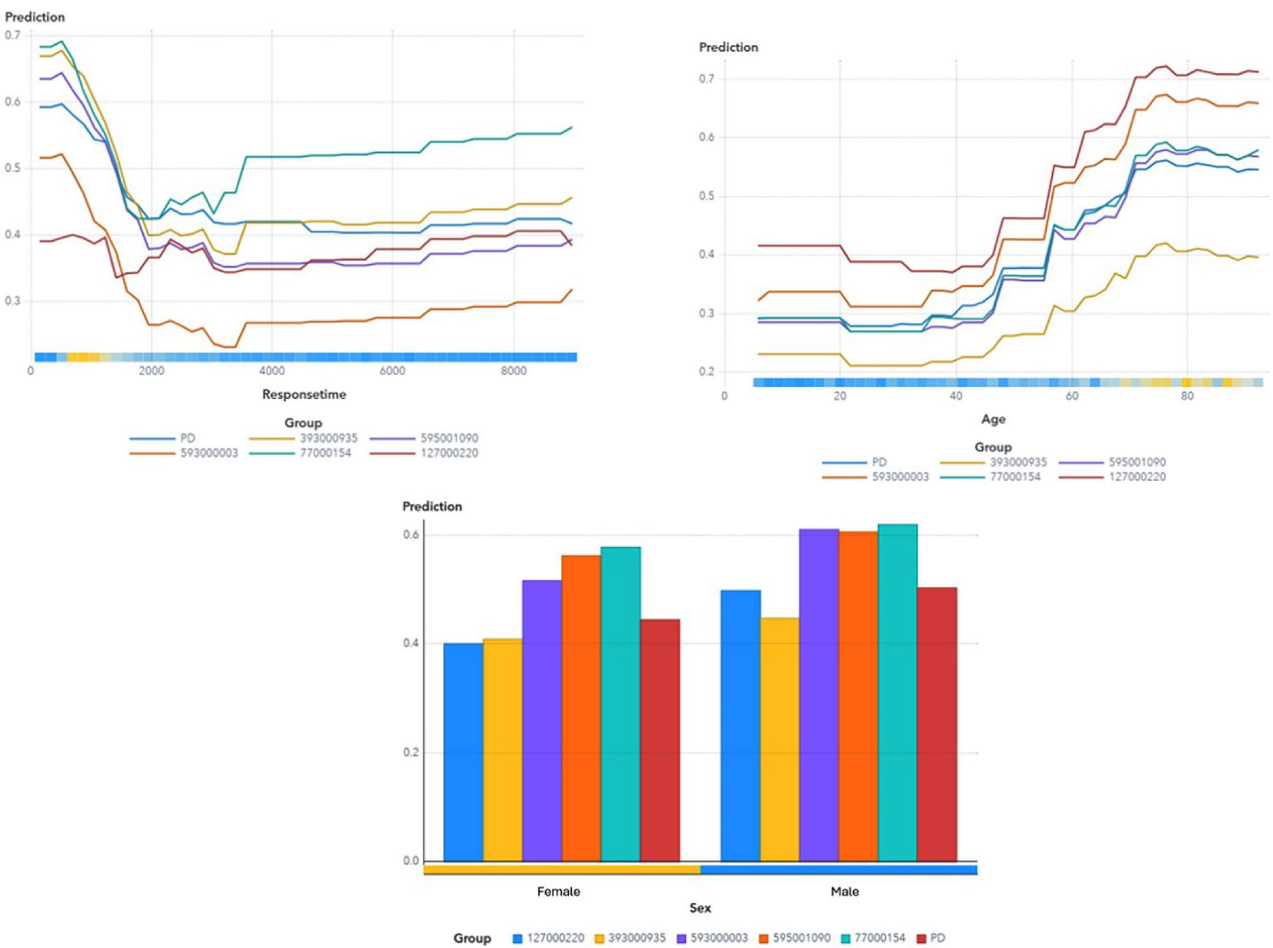
The ICE- plots show the partial dependency (PD) and the relationship between Response time (in seconds), age, and sex on the X-axis, and the Predicted Probability of HRTS on the y-axis for each individual observation. For each observation, the ICE plot displays values of interest on the x-axis and the corresponding prediction for the target variable on the y-axis, holding the other inputs constant at their values for each observation [44]. The group is coded with different identification number for the specific group

Factors influencing the probability of a patient presenting with a time-sensitive condition is illustrated in the ICE plot (Fig. 5) [43]. Shorter response times were generally associated with higher probabilities of identifying patients with HRTS. This makes sense: patients with obvious signs of severe trouble should, in theory, receive faster EMS response. However, as response times became longer, we observed complex, nonlinear patterns. For patients over 60, the likelihood of HRTS started to rise again after about two hours. For younger male patients, the probability of HRTS continued to increase almost linearly with very long response times, again hinting that some serious cases might be missed by initial assessments.

**Fig. 6:**
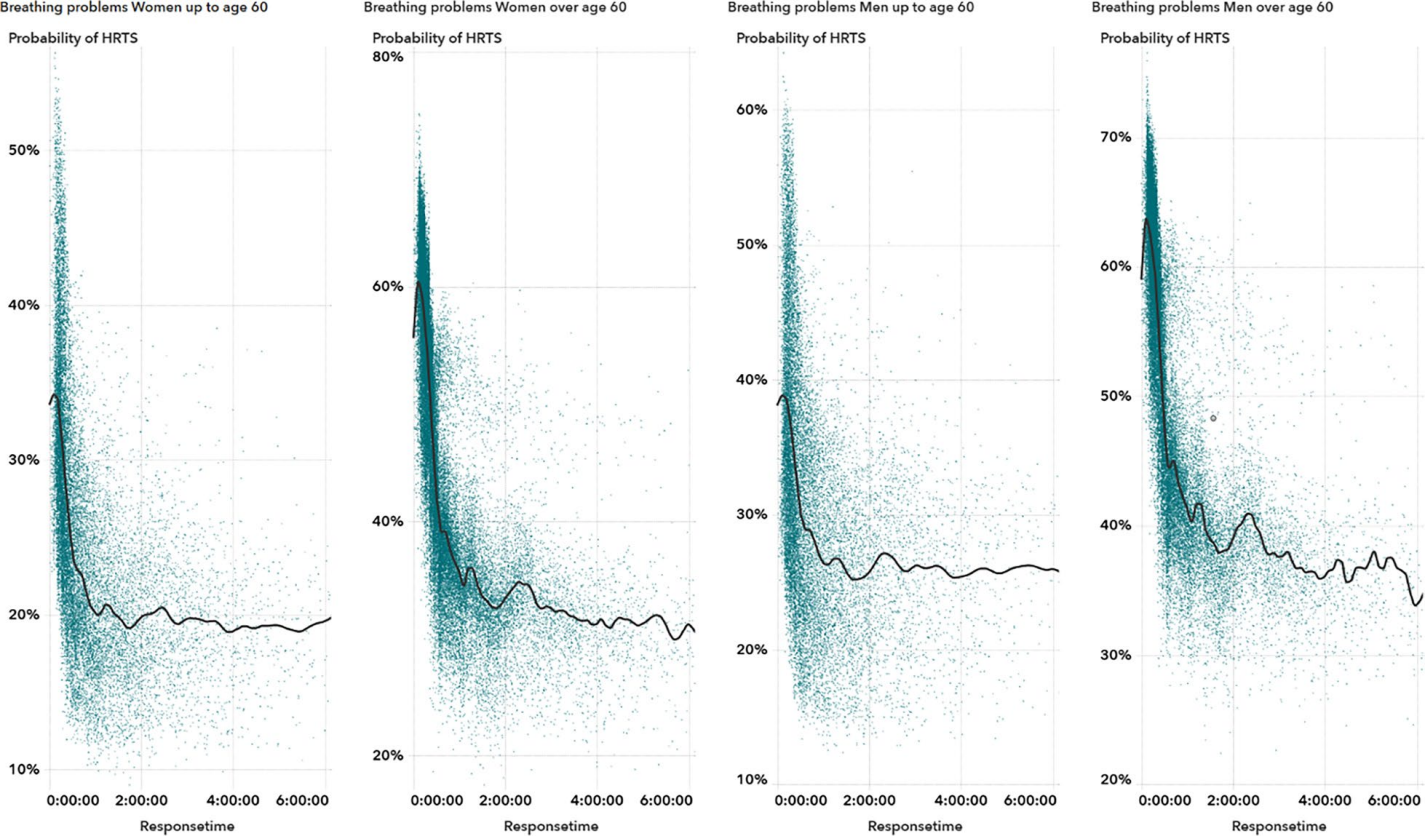
Scatter plots showing the relationship between EMS response time and predicted probability of high-risk, time-sensitive (HRTS) conditions among patients presenting with breathing problems. Panels are stratified by sex (women vs. men) and age group (≤ 60 years vs. >60 years). Each point represents a single observation, and the solid black line indicates a smoothed trend. Notably, high predicted probabilities cluster at shorter response times, while subtle nonlinear patterns emerge at longer delays, varying by both age and sex

When analyzing the interaction of multiple variables using ICE plots and scatterplots, both methods showed an increased patient probability of HRTS as response times lengthen under certain conditions [44]. Specifically, for patients aged over 60, the probability of a time-sensitive condition rises as response times approach approximately two hours, followed by a subsequent nonlinear decrease (Fig. 6).

## Discussion

### Key results

This study establishes an association between prehospital response time and HRTS initially presented with breathing problems at EMCC. Crucially, this work did not aim to produce a high-performing predictive model, as evidenced by the moderate AUC (0.66) and F1-score (0.55). Instead, ML methods were employed to unearth potential nonlinearities—particularly in how the risk of HRTS changes beyond two hours of waiting time, and how age and sex further interact with that risk. The novel approach measures the relationship between response time and the probability of HRTS on scene in the prehospital setting. The model attained a sensitivity of approximately 63% and a specificity of around 61% when predicting HRTS. In contrast, Torlén Wennlund et al. [9] reported a sensitivity of 30% and a specificity of 84.8% for a triage approach in a similar Swedish setting when an emergency call was assessed by an Emergency Medical Dispatcher and a Registered Nurse [9]. These different metrics reflect a fundamental trade-off: higher sensitivity typically reduces the likelihood of missed high-risk cases but may increase the rate of lower-risk calls being classified as high priority. Conversely, a focus on higher specificity leads to fewer false alarms yet can result in more genuinely urgent cases being overlooked.

When deciding which balance is preferable, practical considerations come into play. A system emphasizing sensitivity may be favored if under-triage is deemed riskier or costlier in terms of patient outcomes. On the other hand, a system of high specificity could be more suitable if resources are constrained, as it minimizes diverting ambulances to relatively lower-risk calls. Ultimately, choosing the appropriate threshold depends on the operational priorities and resource limitations inherent in each EMS environment.

A further examination of these findings highlights that each feature response time, age, and sex, uniquely shapes the likelihood of encountering an HRTS condition [8] [10]. The nonlinearity observed with longer response times suggests that some patients, despite receiving an initially lower priority, might have deteriorating conditions that go unnoticed without periodic re-triage [6] [9]. Incorporating a mechanism for systematically checking back with lower-priority calls, especially when waiting periods exceed a certain threshold, could therefore refine resource allocation and prevent delayed identification of critical cases. The significance of age, particularly in individuals over 60, appears linked to physiological vulnerabilities in respiratory function [18] [21] (Fig. 6). Early, more targeted screening questions that assess these risk factors could improve how EMCC personnel recognize high-risk presentations. Sex-based differences also emerged, with male patients consistently more likely to present HRTS; although the precise clinical rationale remains unclear, it underscores a need for EMCC protocols to account for potential risk elevation in men presenting with respiratory complaints. Together, these predictors reveal how a combination of demographic information and evolving response intervals can inform more precise decision-making. Integrating model-generated risk estimates into EMCC workflows may allow dispatchers to adapt on the fly, balancing the urgency of older or male patients who exhibit signs of potential respiratory compromise against other concurrently triaged calls. By weaving these predictor-specific insights into existing training and decision-support protocols, EMCC staff could identify subtle clinical cues earlier, ultimately curbing the impact of prolonged response times on patient outcomes.

### Implications for EMS management

The study findings underscore the importance of integrating response time, age, and sex into EMCC workflows to improve the accuracy of telephone triage. Although traditionally, response time has been seen as a broad indicator of EMS performance, our results reveal complex patterns when delays extend beyond two hours, especially in older adults and certain younger male patients. This insight suggests that initial EMCC priority assignments may require ongoing re-evaluation. Implementing protocols where EMCC personnel revisit calls exceeding a specified waiting interval, particularly for patients whose demographic or clinical profile aligns with high-risk patterns, could help identify deteriorating conditions more promptly and avert missed opportunities for timely intervention.

Incorporating age in triage processes could be done by assigning a higher baseline priority level or additional screening questions for individuals over 60 years. In practice, this may involve training call-takers to probe more deeply into potential respiratory compromise or underlying comorbidities. Such an age-tailored approach could direct advanced life-support units toward older adults who, due to age-related physiological declines, may be at higher risk of severe outcomes if not treated swiftly. Indeed, as noted by Ruge et al. [19] and Malmer et al. [20] under-triage of older patients may contribute to delayed interventions, poorer outcomes, and increased short-term mortality. Incorporating age as a core triage variable in EMCC workflows, potentially with dedicated age-sensitive screening questions, could reduce these avoidable risks, ensure more precise resource allocation, and mitigate adverse effects associated with prolonged waiting times.

Sex was another key predictor, with male patients across all age groups showing a higher probability of HRTS than females. While the exact causal mechanisms remain unclear, EMCC call scripts and dispatcher training could be refined to include prompts that systematically assess risk factors more prevalent in men, such as smoking history or cardiac issues. Implementing a slight increase in vigilance for male callers, especially those with certain symptom descriptions, may help ensure dispatchers do not inadvertently under-triage a subset of patients more prone to critical deterioration.

By embedding these evidence-based modifications into EMCC protocols, healthcare systems can better align ambulance dispatch with the actual risk of time-sensitive respiratory emergencies. This alignment has implications for resource optimization, as it directs finite EMS resources toward patients who stand to benefit the most from shorter response times. Furthermore, enhancing dispatcher awareness of the demographic variables most predictive of HRTS, and training them to respond accordingly, could reduce misclassification rates and lead to improved patient outcomes. Finally, as machine learning-driven systems mature, dynamic re-prioritization could be implemented in real-time, ensuring that changing clinical information or unexpectedly prolonged wait times are promptly recognized and addressed.

### Limitations and future research

While machine learning provides powerful insights, these models can appear as “black boxes” to practitioners. We used PD and ICE plots to improve interpretability; however, more transparent models or inclusion of additional factors, such as socioeconomic status or geographic differences, could further elucidate the mechanisms behind HRTS predictions. Moreover, our directed acyclic graph (DAG) focuses on a limited set of variables (response time, age, and sex). Key details like comorbidities or other clinical data were not included due to data availability and the specific scope of this study, leaving some open pathways and unaccounted confounders. Consequently, these findings may not readily extend beyond this single patient group, those with breathing problems, and one geographic region. Future research should consider a more comprehensive DAG, incorporating a broader range of variables, and investigating whether similar patterns exist in conditions such as cardiac arrest or stroke, as well as in other healthcare systems, to strengthen the causal framework and enhance the robustness of our results.

Red and Orange RETTS triage levels were combined into HRTS. However, it is important to note that Red and Orange RETTS triage levels do not equate to an ‘confirmed’ HRTS but rather that red/orange RETTS represent a probabilistic higher risk for HRTS.

### Generalizability

Our large sample size suggests the results are applicable to other EMS systems with similar healthcare structures and resources. However, local differences, such as variations in healthcare policy or infrastructure, should be considered before generalizing these findings widely.

## Conclusion

By using ML models exploratively, we identified how EMS response time, age, and sex collectively shape the likelihood of encountering HRTS among patients presenting with breathing problems. Our results highlight key risk patterns, particularly for older adults with longer response times, offering hypothesis-generating evidence to refine triage protocols and guide future, more predictive-focused investigations. Ultimately, this study underscores the value of ML for revealing nuanced interactions in EMS data, even when it does not yield a deployable predictive tool.

## Electronic supplementary material

Below is the link to the electronic supplementary material.

Supplementary Material 1

## Data Availability

Due to the sensitive nature of the used data in this study, where data is confidential due to the Swedish Public Access to Information and Secrecy Act. The variables used in the study is reported in supplementary material. It is possible to use the reported variables to request the data from Region Stockholm and their Centre of Health data.

## Declarations

### Adherence to the Declaration of Helsinki

This study adheres to the principles of the Declaration of Helsinki and good clinical practice standards.
