# Supplementary material for: Uncovering nonlinear patterns in time-sensitive prehospital breathing emergencies: an exploratory machine learning study
Source: BMC Med Inform Decis Mak. 2025 Jun 3;25:205. doi: 10.1186/s12911-025-03046-z (PMC12135243; doi:10.1186/s12911-025-03046-z)
Supplement: Supplementary file 1 — Supplementary Material 1 [file 12911_2025_3046_MOESM1_ESM.pdf]

Appendix

A. Features

Feature	Explanation
HRTS (Target)	A patients first assessment by EMS on site with Red or Orange RETTS triage level.
Response time	A created feature using the time difference between when the EMCC call was received, and the first EMS resource arrival at scene of incident. The response time is a result of call handling time and travel time and might influence HTRS.
Avg Response time H	Average Response Time current hour of the observation which is a proxy variable for the level of workload that reflects in this variable and influence response time.
Hour	The hour of day is used to investigate differences in response time between hour of day. Differences related to hour might indicate EMS allocation out of tune and influence response time.
Month	The month of year is used to investigate differences between month of year. Differences related to month might indicate EMS allocation out of tune and influence response time.
Age	Age of the patient might influence HTRS

Table 1: A concise summary of the target (HRTS) and each predictor variable (Response Time, Average Response Time, Hour, Month, Age), including its definition and relevance for assessing prehospital time-sensitive conditions in patients with acute needs.

B. SAS Code

B.1. SAS code from feature engineering in SAS Enterprise Guide 8.2

```
/* ----------------------------------------
Kod exporterad från SAS Enterprise Guide
DATUM: den 5 november 2024     TID: 11:31:51
PROJEKT: Study II 4.0
PROJEKTSÖKVÄG: C:\Users\4khw\OneDrive - Karolinska
Institutet\General\Studie II\Analys\SAS\Study II 4.0.egp
---------------------------------------- */

/* Bibliotekstilldelning för SASAppUser.BI_KFA */
Libname BI_KFA META
LIBURI="SASLibrary?*[@Name='BI_KFA'][DeployedComponents/ServerContex
t[@Name='SASAppUser']]" METAOUT=DATA;
/* Bibliotekstilldelning för SASAppUser.BI_KFA */
Libname BI_KFA META
LIBURI="SASLibrary?*[@Name='BI_KFA'][DeployedComponents/ServerContex
t[@Name='SASAppUser']]" METAOUT=DATA;
/* Bibliotekstilldelning för SASAppUser.BI_KFA */
Libname BI_KFA META
LIBURI="SASLibrary?*[@Name='BI_KFA'][DeployedComponents/ServerContex
t[@Name='SASAppUser']]" METAOUT=DATA;
/* Bibliotekstilldelning för SASAppUser.BI_KFA */
Libname BI_KFA META
LIBURI="SASLibrary?*[@Name='BI_KFA'][DeployedComponents/ServerContex
t[@Name='SASAppUser']]" METAOUT=DATA;
/* Bibliotekstilldelning för SASAppUser.J */
Libname J V9 '/home2/S4KHW' ;

/* Conditionally delete set of tables or views, if they exists
*/
/* If the member does not exist, then no action is performed   */
%macro _eg_conditional_dropds /parmbuff;
```

```
        %local num;
        %local stepneeded;
        %local stepstarted;
        %local dsname;
        %local name;

        %let num=1;
        /* flags to determine whether a PROC SQL step is needed */
        /* or even started yet                                  */
        %let stepneeded=0;
        %let stepstarted=0;
        %let dsname= %qscan(&syspbuff,&num,',()');
        %do %while(&dsname ne);
                %let name = %sysfunc(left(&dsname));
                %if %qsysfunc(exist(&name)) %then %do;
                        %let stepneeded=1;
                        %if (&stepstarted eq 0) %then %do;
                                proc sql;
                                %let stepstarted=1;

                        %end;
                                drop table &name;
                %end;

                %if %sysfunc(exist(&name,view)) %then %do;
                        %let stepneeded=1;
                        %if (&stepstarted eq 0) %then %do;
                                proc sql;
                                %let stepstarted=1;
                        %end;
                                drop view &name;
                %end;
                %let num=%eval(&num+1);
        %let dsname=%qscan(&syspbuff,&num,',()');
        %end;
        %if &stepstarted %then %do;
                quit;
        %end;
%mend _eg_conditional_dropds;


/* Build where clauses from stored process parameters */
%macro _eg_WhereParam( COLUMN, PARM, OPERATOR, TYPE=S,
MATCHALL=_ALL_VALUES_, MATCHALL_CLAUSE=1, MAX= , IS_EXPLICIT=0,
MATCH_CASE=1);

  %local q1 q2 sq1 sq2;
  %local isEmpty;
  %local isEqual isNotEqual;
  %local isIn isNotIn;
  %local isString;
  %local isBetween;

  %let isEqual = ("%QUPCASE(&OPERATOR)" = "EQ" OR "&OPERATOR" =
"=");
```

```
  %let isNotEqual = ("%QUPCASE(&OPERATOR)" = "NE" OR "&OPERATOR" =
"<>");
  %let isIn = ("%QUPCASE(&OPERATOR)" = "IN");
  %let isNotIn = ("%QUPCASE(&OPERATOR)" = "NOT IN");
  %let isString = (%QUPCASE(&TYPE) eq S or %QUPCASE(&TYPE) eq STRING
);
  %if &isString %then
  %do;
        %if "&MATCH_CASE" eq "0" %then %do;
              %let COLUMN = %str(UPPER%(&COLUMN%));
        %end;
        %let q1=%str(%");
        %let q2=%str(%");
        %let sq1=%str(%');
        %let sq2=%str(%');
  %end;
  %else %if %QUPCASE(&TYPE) eq D or %QUPCASE(&TYPE) eq DATE %then
  %do;
    %let q1=%str(%");
    %let q2=%str(%"d);
        %let sq1=%str(%');
    %let sq2=%str(%');
  %end;
  %else %if %QUPCASE(&TYPE) eq T or %QUPCASE(&TYPE) eq TIME %then
  %do;
    %let q1=%str(%");
    %let q2=%str(%"t);
        %let sq1=%str(%');
    %let sq2=%str(%');
  %end;
  %else %if %QUPCASE(&TYPE) eq DT or %QUPCASE(&TYPE) eq DATETIME
%then
  %do;
    %let q1=%str(%");
    %let q2=%str(%"dt);
        %let sq1=%str(%');
    %let sq2=%str(%');
  %end;
  %else
  %do;
    %let q1=;
    %let q2=;
        %let sq1=;
    %let sq2=;
  %end;

  %if "&PARM" = "" %then %let PARM=&COLUMN;

  %let isBetween = ("%QUPCASE(&OPERATOR)"="BETWEEN" or
"%QUPCASE(&OPERATOR)"="NOT BETWEEN");

  %if "&MAX" = "" %then %do;
    %let MAX = &parm._MAX;
    %if &isBetween %then %let PARM = &parm._MIN;
  %end;
```

```
  %if not %symexist(&PARM) or (&isBetween and not %symexist(&MAX))
%then %do;
    %if &IS_EXPLICIT=0 %then %do;
                   not &MATCHALL_CLAUSE
          %end;
          %else %do;
              not 1=1
          %end;
  %end;
  %else %if "%qupcase(&&&PARM)" = "%qupcase(&MATCHALL)" %then %do;
    %if &IS_EXPLICIT=0 %then %do;
              &MATCHALL_CLAUSE
          %end;
          %else %do;
              1=1
          %end;
  %end;
  %else %if (not %symexist(&PARM._count)) or &isBetween %then %do;
    %let isEmpty = ("&&&PARM" = "");
    %if (&isEqual AND &isEmpty AND &isString) %then
       &COLUMN is null;
    %else %if (&isNotEqual AND &isEmpty AND &isString) %then
       &COLUMN is not null;
    %else %do;
             %if &IS_EXPLICIT=0 %then %do;
           &COLUMN &OPERATOR
                            %if "&MATCH_CASE" eq "0" %then %do;

          %unquote(&q1)%QUPCASE(&&&PARM)%unquote(&q2)
                            %end;
                            %else %do;

          %unquote(&q1)&&&PARM%unquote(&q2)
                            %end;
             %end;
             %else %do;
                 &COLUMN &OPERATOR
                            %if "&MATCH_CASE" eq "0" %then %do;

          %unquote(%nrstr(&sq1))%QUPCASE(&&&PARM)%unquote(%nrstr(&sq
2))
                            %end;
                            %else %do;

          %unquote(%nrstr(&sq1))&&&PARM%unquote(%nrstr(&sq2))
                            %end;
             %end;
       %if &isBetween %then
          AND %unquote(&q1)&&&MAX%unquote(&q2);
    %end;
  %end;
  %else
  %do;
          %local emptyList;
          %let emptyList = %symexist(&PARM._count);
          %if &emptyList %then %let emptyList = &&&PARM._count = 0;
```

```
          %if (&emptyList) %then
          %do;
                    %if (&isNotin) %then
                       1;
                    %else
                              0;
          %end;
          %else %if (&&&PARM._count = 1) %then
    %do;
      %let isEmpty = ("&&&PARM" = "");
      %if (&isIn AND &isEmpty AND &isString) %then
        &COLUMN is null;
      %else %if (&isNotin AND &isEmpty AND &isString) %then
        &COLUMN is not null;
      %else %do;
              %if &IS_EXPLICIT=0 %then %do;
                              %if "&MATCH_CASE" eq "0" %then %do;
                                        &COLUMN &OPERATOR
(%unquote(&q1)%QUPCASE(&&&PARM)%unquote(&q2))
                              %end;
                              %else %do;
                                        &COLUMN &OPERATOR
(%unquote(&q1)&&&PARM%unquote(&q2))
                              %end;
              %end;
                    %else %do;
                        &COLUMN &OPERATOR (
                              %if "&MATCH_CASE" eq "0" %then %do;

          %unquote(%nrstr(&sq1))%QUPCASE(&&&PARM)%unquote(%nrstr(&sq
2)))
                              %end;
                              %else %do;

          %unquote(%nrstr(&sq1))&&&PARM%unquote(%nrstr(&sq2)))
                              %end;
                    %end;
            %end;
    %end;
    %else
    %do;
       %local addIsNull addIsNotNull addComma;
       %let addIsNull = %eval(0);
       %let addIsNotNull = %eval(0);
       %let addComma = %eval(0);
       (&COLUMN &OPERATOR (
       %do i=1 %to &&&PARM._count;
          %let isEmpty = ("&&&PARM&i" = "");
          %if (&isString AND &isEmpty AND (&isIn OR &isNotIn)) %then
          %do;
             %if (&isIn) %then %let addIsNull = 1;
             %else %let addIsNotNull = 1;
          %end;
          %else
          %do;
            %if &addComma %then %do;,%end;
```

```
                                %if &IS_EXPLICIT=0 %then %do;
                                          %if "&MATCH_CASE" eq "0"
%then %do;

          %unquote(&q1)%QUPCASE(&&&PARM&i)%unquote(&q2)
                                          %end;
                                          %else %do;

          %unquote(&q1)&&&PARM&i%unquote(&q2)
                                          %end;
                                %end;
                                %else %do;
                                          %if "&MATCH_CASE" eq "0"
%then %do;

          %unquote(%nrstr(&sq1))%QUPCASE(&&&PARM&i)%unquote(%nrstr(&
sq2))
                                          %end;
                                          %else %do;

          %unquote(%nrstr(&sq1))&&&PARM&i%unquote(%nrstr(&sq2))
                                          %end;
                                %end;
            %let addComma = %eval(1);
          %end;
       %end;)
       %if &addIsNull %then OR &COLUMN is null;
       %else %if &addIsNotNull %then AND &COLUMN is not null;
       %do;)
       %end;
    %end;
  %end;
%mend _eg_WhereParam;


/* ---------------------------------- */
/* MACRO: enterpriseguide             */
/* PURPOSE: define a macro variable   */
/*   that contains the file system    */
/*   path of the WORK library on the  */
/*   server.  Note that different     */
/*   logic is needed depending on the */
/*   server type.                     */
/* ---------------------------------- */
%macro enterpriseguide;
%global sasworklocation;
%local tempdsn unique_dsn path;

%if &sysscp=OS %then %do; /* MVS Server */
          %if %sysfunc(getoption(filesystem))=MVS %then %do;
        /* By default, physical file name will be considered a
classic MVS data set. */
              /* Construct dsn that will be unique for each
concurrent session under a particular account: */
                    filename egtemp '&egtemp' disp=(new,delete); /*
create a temporary data set */
```

```
                    %let tempdsn=%sysfunc(pathname(egtemp)); /* get
dsn */
                    filename egtemp clear; /* get rid of data set -
we only wanted its name */
                    %let unique_dsn=".EGTEMP.%substr(&tempdsn, 1,
16).PDSE";
                    filename egtmpdir &unique_dsn
                              disp=(new,delete,delete)
space=(cyl,(5,5,50))
                              dsorg=po dsntype=library recfm=vb
                              lrecl=8000 blksize=8004 ;
                    options fileext=ignore ;
          %end;
          %else %do;
        /*
                    By default, physical file name will be
considered an HFS
                    (hierarchical file system) file.
                    */
                    %if "%sysfunc(getoption(filetempdir))"="" %then
%do;
                              filename egtmpdir '/tmp';
                    %end;
                    %else %do;
                              filename egtmpdir
"%sysfunc(getoption(filetempdir))";
                    %end;
          %end;
          %let path=%sysfunc(pathname(egtmpdir));
    %let sasworklocation=%sysfunc(quote(&path));
%end; /* MVS Server */
%else %do;
          %let sasworklocation = "%sysfunc(getoption(work))/";
%end;
%if &sysscp=VMS_AXP %then %do; /* Alpha VMS server */
          %let sasworklocation = "%sysfunc(getoption(work))";
%end;
%if &sysscp=CMS %then %do;
          %let path = %sysfunc(getoption(work));
          %let sasworklocation = "%substr(&path, %index(&path,%str(
)))";
%end;
%mend enterpriseguide;

%enterpriseguide


/* save the current settings of XPIXELS and YPIXELS */
/* so that they can be restored later               */
%macro _sas_pushchartsize(new_xsize, new_ysize);
          %global _savedxpixels _savedypixels;
          options nonotes;
          proc sql noprint;
          select setting into :_savedxpixels
          from sashelp.vgopt
          where optname eq "XPIXELS";
```

```
            select setting into :_savedypixels
            from sashelp.vgopt
            where optname eq "YPIXELS";
            quit;
            options notes;
            GOPTIONS XPIXELS=&new_xsize YPIXELS=&new_ysize;
%mend _sas_pushchartsize;

/* restore the previous values for XPIXELS and YPIXELS */
%macro _sas_popchartsize;
            %if %symexist(_savedxpixels) %then %do;
                        GOPTIONS XPIXELS=&_savedxpixels
YPIXELS=&_savedypixels;
                        %symdel _savedxpixels / nowarn;
                        %symdel _savedypixels / nowarn;
            %end;
%mend _sas_popchartsize;


ODS PROCTITLE;
OPTIONS DEV=SVG;
GOPTIONS XPIXELS=0 YPIXELS=0;
%macro HTML5AccessibleGraphSupported;
    %if %_SAS_VERCOMP_FV(9,4,4, 0,0,0) >= 0 %then ACCESSIBLE_GRAPH;
%mend;
FILENAME EGHTMLX TEMP;
ODS HTML5(ID=EGHTMLX) FILE=EGHTMLX
    OPTIONS(BITMAP_MODE='INLINE')
    %HTML5AccessibleGraphSupported
    ENCODING='utf-8'
    STYLE=HTMLBlue
    NOGTITLE
    NOGFOOTNOTE
    GPATH=&sasworklocation
;

/*   START PÅ NOD: Frågebyggare-Grundläggande filter   */
%LET _CLIENTTASKLABEL='Frågebyggare-Grundläggande filter';
%LET _CLIENTPROCESSFLOWNAME='Processflöde';
%LET _CLIENTPROJECTPATH='C:\Users\4khw\OneDrive - Karolinska
Institutet\General\Studie II\Analys\SAS\Study II 4.0.egp';
%LET _CLIENTPROJECTPATHHOST='RSTPC00020406';
%LET _CLIENTPROJECTNAME='Study II 4.0.egp';

%_eg_conditional_dropds(WORK.QUERY_FOR_DYNEMSALL_TASKLASTVALU);

PROC SQL;
   CREATE TABLE WORK.QUERY_FOR_DYNEMSALL_TASKLASTVALU AS
   SELECT DISTINCT /* REDORANGE_YELLOWGREEN */
                     (CASE
                        WHEN 1 = t5.RiskLevel THEN 1
                        WHEN 2 = t5.RiskLevel THEN 1
                        WHEN 3 = t5.RiskLevel THEN 2
                        WHEN 4 = t5.RiskLevel THEN 2
                        ELSE t5.RiskLevel
                     END) FORMAT=NUMX12. AS REDORANGE_YELLOWGREEN,
```

```
          t3.journalid_lopnr,
          /* Responsetime */
            (((INPUT(t1.MOTTAGEN,anydtdtm.))-
(input(t1.LARMTIDAMBULANS, anydtdtm.)))+(t2.FirstFramme-
t2.FirstMottaget))
            FORMAT=NUMX8. AS Responsetime,
          t1.incidentid_lopnr,
          t3.IDNR_Lopnr,
          t1.taskid_lopnr,
          /* Date */
            (DATEPART(input(t1.LARMTIDAMBULANS, anydtdtm.)))
FORMAT=AFRDFDE7. AS Date,
          /* MissionCancelled */
            (COUNT(DISTINCT t1.Avbrutet_uppdrag)) FORMAT=NUMX12. AS
MissionCancelled,
          t1.RakelIndex1,
          t2.KOMMUN,
          t5.EssCodeKeywords AS LastEssCodeKeywords,
          t3.KON,
          /* Age */
            ((YEAR(DATEPART((input(t1.LARMTIDAMBULANS,
anydtdtm.)))))-(input(SUBSTR(t3.Fmanad, 1, 4), NUMX4.)))
            FORMAT=NUMX3. AS Age,
          /* Reson of contact to EMCC */
            (CASE
               WHEN '' = t1.RakelIndex1 THEN 'Fever/Infection'
               WHEN '' = t1.RakelIndex1 THEN 'Diarrhea'
               WHEN '' = t1.RakelIndex1 THEN 'Change behavior'
               WHEN '' = t1.RakelIndex1 THEN 'Cardiac arrest'
               WHEN '' = t1.RakelIndex1 THEN 'Fever/Infection'
               WHEN '' = t1.RakelIndex1 THEN 'Minor Trauma/Wound'
               WHEN 'Allergi' = t1.RakelIndex1 THEN 'Allergy'
               WHEN 'Allergi Barn 1-6 år' = t1.RakelIndex1 THEN
'Allergy'
               WHEN 'Allergi Barn 7-15 år' = t1.RakelIndex1 THEN
'Allergy'
               WHEN 'Allergi Spädbarn' = t1.RakelIndex1 THEN
'Allergy'
               WHEN 'Allergi Vuxen' = t1.RakelIndex1 THEN 'Allergy'
               WHEN 'Andningsbesvär' = t1.RakelIndex1 THEN
'Breathing problems'
               WHEN 'Andningsbesvär Barn 1-6 år' = t1.RakelIndex1
THEN 'Breathing problems'
               WHEN 'Andningsbesvär Barn 7-15 år' = t1.RakelIndex1
THEN 'Breathing problems'
               WHEN 'Andningsbesvär Spädbarn' = t1.RakelIndex1 THEN
'Breathing problems'
               WHEN 'Andningsbesvär Vuxen' = t1.RakelIndex1 THEN
'Breathing problems'
               WHEN 'Andningssvårigheter' = t1.RakelIndex1 THEN
'Breathing problems'
               WHEN 'Barn-sjukdom' = t1.RakelIndex1 THEN 'Child
illness'
               WHEN 'Bett/Stick Barn 1-6 år' = t1.RakelIndex1 THEN
'Insect bite'
```

```
                WHEN 'Bett/Stick Vuxen' = t1.RakelIndex1 THEN 'Insect
bite'
                WHEN 'Blödning Ansikte' = t1.RakelIndex1 THEN
'Bleeding'
                WHEN 'Blödning Ansikte Vuxen' = t1.RakelIndex1 THEN
'Bleeding'
                WHEN 'Blödning Barn 1-6 år' = t1.RakelIndex1 THEN
'Bleeding'
                WHEN 'Blödning Barn 7-15 år' = t1.RakelIndex1 THEN
'Bleeding'
                WHEN 'Blödning Barn 7-15år' = t1.RakelIndex1 THEN
'Bleeding'
                WHEN 'Blödning Bål Vuxen' = t1.RakelIndex1 THEN
'Bleeding'
                WHEN 'Blödning Extremitet' = t1.RakelIndex1 THEN
'Bleeding'
                WHEN 'Blödning Extremitet Vuxen' = t1.RakelIndex1
THEN 'Bleeding'
                WHEN 'Blödning Mun' = t1.RakelIndex1 THEN 'Bleeding'
                WHEN 'Blödning Mun Vuxen' = t1.RakelIndex1 THEN
'Bleeding'
                WHEN 'Blödning Näsa' = t1.RakelIndex1 THEN 'Bleeding'
                WHEN 'Blödning Näsa Vuxen' = t1.RakelIndex1 THEN
'Bleeding'
                WHEN 'Blödning Rektal' = t1.RakelIndex1 THEN
'Bleeding'
                WHEN 'Blödning Rektal Vuxen' = t1.RakelIndex1 THEN
'Bleeding'
                WHEN 'Blödning Urinväg' = t1.RakelIndex1 THEN
'Bleeding'
                WHEN 'Blödning Urinväg Vuxen' = t1.RakelIndex1 THEN
'Bleeding'
                WHEN 'Blödning Vaginal' = t1.RakelIndex1 THEN
'Bleeding'
                WHEN 'Blödning Vaginal Vuxen' = t1.RakelIndex1 THEN
'Bleeding'
                WHEN 'Blödning. ej trauma' = t1.RakelIndex1 THEN
'Bleeding'
                WHEN 'Brännskada' = t1.RakelIndex1 THEN 'Burn'
                WHEN 'Brännskada Barn 7-15år' = t1.RakelIndex1 THEN
'Burn'
                WHEN 'Brännskada Vuxen' = t1.RakelIndex1 THEN 'Burn'
                WHEN 'Brännskada/Elolycka' = t1.RakelIndex1 THEN
'Burn'
                WHEN 'Brännskada/Elolycka Barn 1-6 år' =
t1.RakelIndex1 THEN 'Burn'
                WHEN 'Brännskada/Elskada' = t1.RakelIndex1 THEN
'Burn'
                WHEN 'Bröstsmärta/Hjärtsjukdom' = t1.RakelIndex1 THEN
'Chest pain'
                WHEN 'Bröstsmärtor/Hjärtsjukdom' = t1.RakelIndex1
THEN 'Chest pain'
                WHEN 'Buk/urinvägar' = t1.RakelIndex1 THEN 'Abdominal
pain/Urinal problem'
                WHEN 'Diarré/Kräkning' = t1.RakelIndex1 THEN
'Diarrhea'
```

```
                    WHEN 'Diarré/Kräkning Barn 1-6 år' = t1.RakelIndex1
THEN 'Diarrhea'
                    WHEN 'Diarré/Kräkning Barn 7-15 år' = t1.RakelIndex1
THEN 'Diarrhea'
                    WHEN 'Diarré/Kräkning Spädbarn' = t1.RakelIndex1 THEN
'Diarrhea'
                    WHEN 'Diarré/Kräkning Vuxen' = t1.RakelIndex1 THEN
'Diarrhea'
                    WHEN 'Extremitet/Sårskador/Mindre trauma' =
t1.RakelIndex1 THEN 'Minor Trauma/Wound'
                    WHEN 'Feber' = t1.RakelIndex1 THEN 'Fever/Infection'
                    WHEN 'Feber Barn 7-15 år' = t1.RakelIndex1 THEN
'Fever/Infection'
                    WHEN 'Feber Vuxen' = t1.RakelIndex1 THEN
'Fever/Infection'
                    WHEN 'Feber/Infektion' = t1.RakelIndex1 THEN
'Fever/Infection'
                    WHEN 'Förgiftning' = t1.RakelIndex1 THEN 'Poisoning'
                    WHEN 'Förgiftning Barn 1-6 år' = t1.RakelIndex1 THEN
'Poisoning'
                    WHEN 'Förgiftning Barn 7-15 år' = t1.RakelIndex1 THEN
'Poisoning'
                    WHEN 'Förgiftning Barn 7-15år' = t1.RakelIndex1 THEN
'Poisoning'
                    WHEN 'Förgiftning Vuxen' = t1.RakelIndex1 THEN
'Poisoning'
                    WHEN 'Förgiftning, överdos' = t1.RakelIndex1 THEN
'Poisoning'
                    WHEN 'Förändrat beteende' = t1.RakelIndex1 THEN
'Change behavior'
                    WHEN 'Förändrat beteende Barn 1-6 år' =
t1.RakelIndex1 THEN 'Change behavior'
                    WHEN 'Förändrat beteende Barn 7-15 år' =
t1.RakelIndex1 THEN 'Change behavior'
                    WHEN 'Förändrat beteende Spädbarn' = t1.RakelIndex1
THEN 'Change behavior'
                    WHEN 'Förändrat beteende Vuxen' = t1.RakelIndex1 THEN
'Change behavior'
                    WHEN 'Hjärtstopp' = t1.RakelIndex1 THEN 'Cardiac
arrest'
                    WHEN 'Hjärtstopp Barn 1-6 år' = t1.RakelIndex1 THEN
'Cardiac arrest'
                    WHEN 'Hjärtstopp Spädbarn' = t1.RakelIndex1 THEN
'Cardiac arrest'
                    WHEN 'Hjärtstopp Vuxen' = t1.RakelIndex1 THEN
'Cardiac arrest'
                    WHEN 'Huvudvärk. Yrsel' = t1.RakelIndex1 THEN 'Minor
Trauma/Wound'
                    WHEN 'Infektion' = t1.RakelIndex1 THEN
'Fever/Infection'
                    WHEN 'Infektion Barn 1-6 år' = t1.RakelIndex1 THEN
'Fever/Infection'
                    WHEN 'Infektion Spädbarn' = t1.RakelIndex1 THEN
'Fever/Infection'
                    WHEN 'Infektion/Misstanke om Sepsis Barn 7-15 år' =
t1.RakelIndex1 THEN 'Fever/Infection'
```

```
                WHEN 'Infektion/Misstanke om Sepsis Vuxen' =
t1.RakelIndex1 THEN 'Fever/Infection'
                WHEN 'Krampanfall' = t1.RakelIndex1 THEN 'Convulsion'
                WHEN 'Kramper/Epilepsi' = t1.RakelIndex1 THEN
'Convulsion'
                WHEN 'Kramper/Epilepsi Barn 1-6 år' = t1.RakelIndex1
THEN 'Convulsion'
                WHEN 'Kramper/Epilepsi Barn 7-15 år' = t1.RakelIndex1
THEN 'Convulsion'
                WHEN 'Kramper/Epilepsi Barn 7-15år' = t1.RakelIndex1
THEN 'Convulsion'
                WHEN 'Kramper/Epilepsi Spädbarn' = t1.RakelIndex1
THEN 'Convulsion'
                WHEN 'Kramper/Epilepsi Vuxen' = t1.RakelIndex1 THEN
'Convulsion'
                WHEN 'Medvetandesänkt' = t1.RakelIndex1 THEN
'Decreased conciousness'
                WHEN 'Medvetandesänkt Barn 1-6 år' = t1.RakelIndex1
THEN 'Decreased conciousness'
                WHEN 'Medvetandesänkt Barn 7-15 år' = t1.RakelIndex1
THEN 'Decreased conciousness'
                WHEN 'Medvetandesänkt Spädbarn' = t1.RakelIndex1 THEN
'Decreased conciousness'
                WHEN 'Medvetandesänkt Vuxen' = t1.RakelIndex1 THEN
'Decreased conciousness'
                WHEN 'Medvetslös' = t1.RakelIndex1 THEN 'Decreased
conciousness'
                WHEN 'Medvetslös Barn 1-6 år' = t1.RakelIndex1 THEN
'Decreased conciousness'
                WHEN 'Medvetslös Barn 7-15 år' = t1.RakelIndex1 THEN
'Decreased conciousness'
                WHEN 'Medvetslös Spädbarn' = t1.RakelIndex1 THEN
'Decreased conciousness'
                WHEN 'Medvetslös vuxen' = t1.RakelIndex1 THEN
'Decreased conciousness'
                WHEN 'Medvetslös Vuxen' = t1.RakelIndex1 THEN
'Decreased conciousness'
                WHEN 'Medvetslös-barn' = t1.RakelIndex1 THEN
'Decreased conciousness'
                WHEN 'Medvetslös-vuxen' = t1.RakelIndex1 THEN
'Decreased conciousness'
                WHEN 'Mindre trauma (lågenergi)' = t1.RakelIndex1
THEN 'Minor Trauma/Wound'
                WHEN 'Mindre trauma (lågenergi) Barn 1-6 år' =
t1.RakelIndex1 THEN 'Minor Trauma/Wound'
                WHEN 'Mindre trauma (lågenergi) Barn 7-15 år' =
t1.RakelIndex1 THEN 'Minor Trauma/Wound'
                WHEN 'Mindre trauma (lågenergi) Barn 7-15år' =
t1.RakelIndex1 THEN 'Minor Trauma/Wound'
                WHEN 'Mindre trauma (lågenergi) Spädbarn' =
t1.RakelIndex1 THEN 'Minor Trauma/Wound'
                WHEN 'Mindre trauma (lågenergi) Vuxen' =
t1.RakelIndex1 THEN 'Minor Trauma/Wound'
                WHEN 'Misstanke om Sepsis' = t1.RakelIndex1 THEN
'Fever/Infection'
```

```
                WHEN 'Misstanke om stroke/TIA' = t1.RakelIndex1 THEN 
'Stroke'
                WHEN 'Misstanke om Stroke/TIA' = t1.RakelIndex1 THEN 
'Stroke'
                WHEN 'Misstanke om Stroke/TIA Vuxen' = t1.RakelIndex1 
THEN 'Stroke'
                WHEN 'Oklart vårdbehov' = t1.RakelIndex1 THEN 
'Uncertain need of care'
                WHEN 'Oklart vårdbehov Barn 1-6 år' = t1.RakelIndex1 
THEN 'Uncertain need of care'
                WHEN 'Oklart vårdbehov Barn 7-15 år' = t1.RakelIndex1 
THEN 'Uncertain need of care'
                WHEN 'Oklart vårdbehov Spädbarn' = t1.RakelIndex1 
THEN 'Uncertain need of care'
                WHEN 'Oklart vårdbehov Vuxen' = t1.RakelIndex1 THEN 
'Uncertain need of care'
                WHEN 'Osäkra uppgifter/Svårt sjuk patient' = 
t1.RakelIndex1 THEN 'Uncertain need of care'
                WHEN 'Ryggbesvär' = t1.RakelIndex1 THEN 'Backpain'
                WHEN 'Slaganfall (Stroke)-förlamningar' = 
t1.RakelIndex1 THEN 'Stroke'
                WHEN 'Stroke -Förlamningar' = t1.RakelIndex1 THEN 
'Stroke'
                ELSE t1.RakelIndex1
             END) FORMAT=$CHAR18. AS 'Reson of contact to EMCC'n,
          /* Hour */
            (HOUR((input(t1.LARMTIDAMBULANS, anydtdtm.)))) 
FORMAT=NUMX12. AS Hour,
          /* Month */
            (MONTH(DATEPART((input(t1.LARMTIDAMBULANS, 
anydtdtm.))))) FORMAT=NUMX12. AS Month,
          t1.PRIORITETUT
      FROM BI_KFA.DYNEMSALL_JOURNALRETTSLAST t5
           RIGHT JOIN (BI_KFA.DYNEMSALL_JOURNALLASTVALUE t3
           LEFT JOIN BI_KFA.DYNEMSALL_TASKLASTVALUE t1 ON 
(t3.taskid_lopnr = t1.taskid_lopnr)) ON (t5.journalid_lopnr = 
          t3.journalid_lopnr)
           LEFT JOIN BI_KFA.DYNEMSALL_INCIDENTLASTVALUE t2 ON 
(t3.incidentid_lopnr = t2.incidentid_lopnr)
      WHERE t1.RakelIndex1 NOT IN 
           (
           'Sekundäruppdrag',
           'Sekundäruppdrag Barn 1-6 år',
           'Sekundäruppdrag Barn 7-15 år',
           'Sekundäruppdrag Spädbarn',
           'Sekundäruppdrag Vuxen',
           'Sjuktransporter',
           'Beställt uppdrag mellan vårdenheter'
           ) AND t1.RakelIndex2 NOT IN 
           (
           'Transport',
           'Transport mellan vårdenheter',
           'Transport till/från vårdenhet',
           'Transport till/från vårdenhet (DOLD)',
           'Transportuppdrag'
```

```
            ) AND t1.EmsUnitType = 'Akutambulans' AND (CALCULATED
Responsetime) BETWEEN 0 AND 36000
      GROUP BY (CALCULATED REDORANGE_YELLOWGREEN),
               t3.journalid_lopnr,
               (CALCULATED Responsetime),
               t1.incidentid_lopnr,
               t3.IDNR_Lopnr,
               t1.taskid_lopnr,
               (CALCULATED Date),
               t1.RakelIndex1,
               t2.KOMMUN,
               t5.EssCodeKeywords,
               t3.KON,
               ((YEAR(DATEPART((input(t1.LARMTIDAMBULANS,
anydtdtm.)))))-(YEAR((INPUT(t3.Fmanad,ddmmyy8.))))),
               (CALCULATED 'Reson of contact to EMCC'n),
               (CALCULATED Hour),
               (CALCULATED Month),
               t1.PRIORITETUT
      ORDER BY t3.journalid_lopnr;
QUIT;




%LET _CLIENTTASKLABEL=;
%LET _CLIENTPROCESSFLOWNAME=;
%LET _CLIENTPROJECTPATH=;
%LET _CLIENTPROJECTPATHHOST=;
%LET _CLIENTPROJECTNAME=;


/*   START PÅ NOD: Frågebyggare   */
%LET _CLIENTTASKLABEL='Frågebyggare';
%LET _CLIENTPROCESSFLOWNAME='Processflöde';
%LET _CLIENTPROJECTPATH='C:\Users\4khw\OneDrive - Karolinska
Institutet\General\Studie II\Analys\SAS\Study II 4.0.egp';
%LET _CLIENTPROJECTPATHHOST='RSTPC00020406';
%LET _CLIENTPROJECTNAME='Study II 4.0.egp';

%_eg_conditional_dropds(WORK.QUERY_FOR_DYNEMSALL_TASKLAS_0000);

PROC SQL;
   CREATE TABLE WORK.QUERY_FOR_DYNEMSALL_TASKLAS_0000 AS
   SELECT t1.Date,
          t1.Hour,
          /* AvgResponsetimeH */
            (AVG(t1.Responsetime)) FORMAT=NUMX8. AS AvgResponsetimeH
      FROM WORK.QUERY_FOR_DYNEMSALL_TASKLASTVALU t1
      WHERE t1.PRIORITETUT = '2'
      GROUP BY t1.Date,
               t1.Hour;
QUIT;
```

```
%LET _CLIENTTASKLABEL=;
%LET _CLIENTPROCESSFLOWNAME=;
%LET _CLIENTPROJECTPATH=;
%LET _CLIENTPROJECTPATHHOST=;
%LET _CLIENTPROJECTNAME=;


/*   START PÅ NOD: Frågebyggare 1   */
%LET _CLIENTTASKLABEL='Frågebyggare 1';
%LET _CLIENTPROCESSFLOWNAME='Processflöde';
%LET _CLIENTPROJECTPATH='C:\Users\4khw\OneDrive - Karolinska
Institutet\General\Studie II\Analys\SAS\Study II 4.0.egp';
%LET _CLIENTPROJECTPATHHOST='RSTPC00020406';
%LET _CLIENTPROJECTNAME='Study II 4.0.egp';

%_eg_conditional_dropds(J.VA_DEMSII);

PROC SQL;
   CREATE TABLE J.VA_DEMSII AS
   SELECT t1.REDORANGE_YELLOWGREEN,
          t1.journalid_lopnr,
          t1.Age,
          t1.Responsetime FORMAT=NUMX12. AS Responsetime,
          t1.taskid_lopnr,
          t1.KON LABEL="Sex" AS Sex,
          t1.'Reson of contact to EMCC'n,
          t1.Month,
          t1.Hour,
          t2.AvgResponsetimeH
      FROM WORK.QUERY_FOR_DYNEMSALL_TASKLASTVALU t1
           LEFT JOIN WORK.QUERY_FOR_DYNEMSALL_TASKLAS_0000 t2 ON
(t1.Date = t2.Date) AND (t1.Hour = t2.Hour);
QUIT;




%LET _CLIENTTASKLABEL=;
%LET _CLIENTPROCESSFLOWNAME=;
%LET _CLIENTPROJECTPATH=;
%LET _CLIENTPROJECTPATHHOST=;
%LET _CLIENTPROJECTNAME=;


/*   START PÅ NOD: Partition Data   */
%LET _CLIENTTASKLABEL='Partition Data';
%LET _CLIENTPROCESSFLOWNAME='Processflöde';
%LET _CLIENTPROJECTPATH='C:\Users\4khw\OneDrive - Karolinska
Institutet\General\Studie II\Analys\SAS\Study II 4.0.egp';
%LET _CLIENTPROJECTPATHHOST='RSTPC00020406';
%LET _CLIENTPROJECTNAME='Study II 4.0.egp';

%macro web_drop_table / parmbuff;%mend;
%macro web_open_table / parmbuff;%mend;
```

```
ODS GRAPHICS ON;
TITLE;FOOTNOTE;
FOOTNOTE1 "Genererat av SAS (&_SASSERVERNAME, &SYSSCPL)
%TRIM(%QSYSFUNC(DATE(), NLDATE20.)) %TRIM(%QSYSFUNC(TIME(),
NLTIME.))";

data _null_;
         idMaxLength=length("Training");
         idMaxLength=max(idMaxLength, length("Validation"));
         idMaxLength=max(idMaxLength, length("Test"));

         /* Put it in a macro variable for use in the real code */
         call symput('idLength', idMaxLength);
run;

proc sql noprint;
         select count(*) into :count from J.VA_DEMSII;
quit;

data J.VA_DEMSIIP;
         set J.VA_DEMSII;
         length _Partition_ $ &idLength;
         retain __tmp1-__tmp%trim(&count) __nobs__ __nobs1__
__nobs2__ __nobs3__;
         drop _i_ __seed__ __tmp1-__tmp%trim(&count);
         drop _n1_ __nobs__ __nobs1__ __nobs2__ __nobs3__;
         array __tmp(*) __tmp1-__tmp%trim(&count);

         if (_n_=1) then
                  do;
                           __seed__=-1;
                           __nobs__=&count;

                           do _i_=1 to dim(__tmp);
                                    __tmp(_i_)=_i_;
                           end;
                           call ranperm(__seed__, of __tmp(*));
                           __nobs1__=round(0.6*__nobs__);

         __nobs2__=round(0.3*__nobs__)+__nobs1__;

         __nobs3__=round(0.1*__nobs__)+__nobs2__;
                  end;
         _n1_=_n_;

         if (_n1_ <=dim(__tmp)) then
                  do;

                           if (__tmp(_n1_) > 0) then
                                    do;

                                                      if (__tmp(_n1_)
<=__nobs1__) then
                                                               do;
```

```
        _Partition_="Training";

        output;
                                                    end;
                                            else if
(__tmp(_n1_) <=__nobs2__) then
                                                    do;

        _Partition_="Validation";

        output;
                                                    end;
                                            else if
(__tmp(_n1_) <=__nobs3__) then
                                                    do;

        _Partition_="Test";

        output;
                                                    end;
                                    end;
                    end;
run;


TITLE;FOOTNOTE;



%LET _CLIENTTASKLABEL=;
%LET _CLIENTPROCESSFLOWNAME=;
%LET _CLIENTPROJECTPATH=;
%LET _CLIENTPROJECTPATHHOST=;
%LET _CLIENTPROJECTNAME=;

;*';*";*/;quit;run;
ODS _ALL_ CLOSE;
```

B.2. SAS code from analyses in SAS Model Studio 8.5

Score code

```
data sasep.out;
   dcl package score _B7C04JZNYHPNOS5YX3FFLGFR3();
   dcl double "P_REDORANGE_YELLOWGREEN1" having label
n'Predicted: REDORANGE_YELLOWGREEN=1';
   dcl double "P_REDORANGE_YELLOWGREEN2" having label
n'Predicted: REDORANGE_YELLOWGREEN=2';
   dcl nchar(32) "I_REDORANGE_YELLOWGREEN" having label
n'Into: REDORANGE_YELLOWGREEN';
   dcl nchar(4) "_WARN_" having label n'Warnings';
   dcl double EM_EVENTPROBABILITY;
   dcl nchar(8) EM_CLASSIFICATION;
   dcl double EM_PROBABILITY;
```

```
   varlist allvars [_all_];


   method init();
      _B7C04JZNYHPNOS5YX3FFLGFR3.setvars(allvars);

_B7C04JZNYHPNOS5YX3FFLGFR3.setkey(n'EEB99AC6A47D0B20597322B7F0
92ABBEAE1B1CB4');
   end;

   method post_B7C04JZNYHPNOS5YX3FFLGFR3();
      dcl double _P_;

      if "P_REDORANGE_YELLOWGREEN1" = . then
"P_REDORANGE_YELLOWGREEN1" = 0.4704105781;
      if "P_REDORANGE_YELLOWGREEN2" = . then
"P_REDORANGE_YELLOWGREEN2" = 0.5295894219;
      if MISSING("I_REDORANGE_YELLOWGREEN") then do ;
      _P_ = 0.0;
      if "P_REDORANGE_YELLOWGREEN2" > _P_ then do ;
      _P_ = "P_REDORANGE_YELLOWGREEN2";
      "I_REDORANGE_YELLOWGREEN" = '2';
      end;
      if "P_REDORANGE_YELLOWGREEN1" > _P_ then do ;
      _P_ = "P_REDORANGE_YELLOWGREEN1";
      "I_REDORANGE_YELLOWGREEN" = '1';
      end;
      end;
      if "P_REDORANGE_YELLOWGREEN2" > 0.6 then do ;
      "I_REDORANGE_YELLOWGREEN" = '2';
      end;
      else do ;
      "I_REDORANGE_YELLOWGREEN" = '1';
      end;
      EM_EVENTPROBABILITY = "P_REDORANGE_YELLOWGREEN1";
      EM_CLASSIFICATION = "I_REDORANGE_YELLOWGREEN";
      EM_PROBABILITY = MAX("P_REDORANGE_YELLOWGREEN1",
"P_REDORANGE_YELLOWGREEN2");

   end;


   method run();
      set SASEP.IN;
      _B7C04JZNYHPNOS5YX3FFLGFR3.scoreRecord();
      post_B7C04JZNYHPNOS5YX3FFLGFR3();
   end;

   method term();
   end;
```

```
enddata;
```

DS2Package code

```
package MS_bd3e7ac5b11d441e8ccac7ea6d7b34bf_02DEC2024082230974
/ overwrite=yes;
   dcl package score _B7C04JZNYHPNOS5YX3FFLGFR3();
   dcl double "P_REDORANGE_YELLOWGREEN1" having label
n'Predicted: REDORANGE_YELLOWGREEN=1';
   dcl double "P_REDORANGE_YELLOWGREEN2" having label
n'Predicted: REDORANGE_YELLOWGREEN=2';
   dcl nchar(32) "I_REDORANGE_YELLOWGREEN" having label
n'Into: REDORANGE_YELLOWGREEN';
   dcl nchar(4) "_WARN_" having label n'Warnings';
   dcl double EM_EVENTPROBABILITY;
   dcl nchar(8) EM_CLASSIFICATION;
   dcl double EM_PROBABILITY;
   varlist allvars [_all_];


   method init();
      _B7C04JZNYHPNOS5YX3FFLGFR3.setvars(allvars);

_B7C04JZNYHPNOS5YX3FFLGFR3.setkey(n'EEB99AC6A47D0B20597322B7F0
92ABBEAE1B1CB4');

_B7C04JZNYHPNOS5YX3FFLGFR3.setOption('_destroyFirstInstLast_',
0);
   end;

   method post_B7C04JZNYHPNOS5YX3FFLGFR3();
      dcl double _P_;

      if "P_REDORANGE_YELLOWGREEN1" = . then
"P_REDORANGE_YELLOWGREEN1" = 0.4704105781;
      if "P_REDORANGE_YELLOWGREEN2" = . then
"P_REDORANGE_YELLOWGREEN2" = 0.5295894219;
      if MISSING("I_REDORANGE_YELLOWGREEN") then do ;
      _P_ = 0.0;
      if "P_REDORANGE_YELLOWGREEN2" > _P_ then do ;
      _P_ = "P_REDORANGE_YELLOWGREEN2";
      "I_REDORANGE_YELLOWGREEN" = '2';
      end;
      if "P_REDORANGE_YELLOWGREEN1" > _P_ then do ;
      _P_ = "P_REDORANGE_YELLOWGREEN1";
      "I_REDORANGE_YELLOWGREEN" = '1';
      end;
      end;
      if "P_REDORANGE_YELLOWGREEN2" > 0.6 then do ;
      "I_REDORANGE_YELLOWGREEN" = '2';
```

```
      end;
      else do ;
      "I_REDORANGE_YELLOWGREEN" = '1';
      end;
      EM_EVENTPROBABILITY = "P_REDORANGE_YELLOWGREEN1";
      EM_CLASSIFICATION = "I_REDORANGE_YELLOWGREEN";
      EM_PROBABILITY = MAX("P_REDORANGE_YELLOWGREEN1",
"P_REDORANGE_YELLOWGREEN2");

   end;


   method score(
      double "Age",
      double "AvgResponsetimeH",
      double "Hour",
      double "journalid_lopnr",
      double "Month",
      double "Responsetime",
      char(12) "Sex",
      double "taskid_lopnr",
      IN_OUT char(32) "EM_CLASSIFICATION",
      IN_OUT double "EM_EVENTPROBABILITY",
      IN_OUT double "EM_PROBABILITY",
      IN_OUT char(32) "I_REDORANGE_YELLOWGREEN",
      IN_OUT double "P_REDORANGE_YELLOWGREEN1",
      IN_OUT double "P_REDORANGE_YELLOWGREEN2",
      IN_OUT double "_P_",
      IN_OUT char(4) "_WARN_"
   );
      this."Age"= "Age";
      this."AvgResponsetimeH"= "AvgResponsetimeH";
      this."Hour"= "Hour";
      this."journalid_lopnr"= "journalid_lopnr";
      this."Month"= "Month";
      this."Responsetime"= "Responsetime";
      this."Sex"= "Sex";
      this."taskid_lopnr"= "taskid_lopnr";

      _B7C04JZNYHPNOS5YX3FFLGFR3.scoreRecord();
      post_B7C04JZNYHPNOS5YX3FFLGFR3();

      "EM_CLASSIFICATION"= this."EM_CLASSIFICATION";
      "EM_EVENTPROBABILITY"= this."EM_EVENTPROBABILITY";
      "EM_PROBABILITY"= this."EM_PROBABILITY";
      "I_REDORANGE_YELLOWGREEN"=
this."I_REDORANGE_YELLOWGREEN";
      "P_REDORANGE_YELLOWGREEN1"=
this."P_REDORANGE_YELLOWGREEN1";
      "P_REDORANGE_YELLOWGREEN2"=
this."P_REDORANGE_YELLOWGREEN2";
```

```
      "_P_"= this."_P_";
      "_WARN_"= this."_WARN_";
   end;


   method predictedscore(
      double "Age",
      double "AvgResponsetimeH",
      double "Hour",
      double "journalid_lopnr",
      double "Month",
      double "Responsetime",
      char(12) "Sex",
      double "taskid_lopnr",
      IN_OUT char(32) "EM_CLASSIFICATION",
      IN_OUT double "EM_EVENTPROBABILITY",
      IN_OUT double "EM_PROBABILITY",
      IN_OUT char(32) "I_REDORANGE_YELLOWGREEN",
      IN_OUT double "P_REDORANGE_YELLOWGREEN1",
      IN_OUT double "P_REDORANGE_YELLOWGREEN2",
      IN_OUT char(4) "_WARN_"
   );
      this."Age"= "Age";
      this."AvgResponsetimeH"= "AvgResponsetimeH";
      this."Hour"= "Hour";
      this."journalid_lopnr"= "journalid_lopnr";
      this."Month"= "Month";
      this."Responsetime"= "Responsetime";
      this."Sex"= "Sex";
      this."taskid_lopnr"= "taskid_lopnr";

      _B7C04JZNYHPNOS5YX3FFLGFR3.scoreRecord();
      post_B7C04JZNYHPNOS5YX3FFLGFR3();

      "EM_CLASSIFICATION"= this."EM_CLASSIFICATION";
      "EM_EVENTPROBABILITY"= this."EM_EVENTPROBABILITY";
      "EM_PROBABILITY"= this."EM_PROBABILITY";
      "I_REDORANGE_YELLOWGREEN"=
this."I_REDORANGE_YELLOWGREEN";
      "P_REDORANGE_YELLOWGREEN1"=
this."P_REDORANGE_YELLOWGREEN1";
      "P_REDORANGE_YELLOWGREEN2"=
this."P_REDORANGE_YELLOWGREEN2";
      "_WARN_"= this."_WARN_";
   end;

endpackage;
```

Training code

```
*------------------------------------------------------------
*;
* Macro Variables for input, output data and files;
  %let dm_datalib =;
  %let dm_lib     = WORK;
  %let dm_folder  = %sysfunc(pathname(work));
*------------------------------------------------------------
*;
*------------------------------------------------------------
*;
  * Training for gradboost;
*------------------------------------------------------------
*;
*------------------------------------------------------------
*;
  * Initializing Variable Macros;
*------------------------------------------------------------
*;
%macro dm_unary_input;
%mend dm_unary_input;
%global dm_num_unary_input;
%let dm_num_unary_input = 0;
%macro dm_interval_input;
   'Age'n 'AvgResponsetimeH'n 'Hour'n 'Month'n 'Responsetime'n
%mend dm_interval_input;
%global dm_num_interval_input;
%let dm_num_interval_input = 5 ;
%macro dm_binary_input;
   'Sex'n
%mend dm_binary_input;
%global dm_num_binary_input;
%let dm_num_binary_input = 1 ;
%macro dm_nominal_input;
%mend dm_nominal_input;
%global dm_num_nominal_input;
%let dm_num_nominal_input = 0;
%macro dm_ordinal_input;
%mend dm_ordinal_input;
%global dm_num_ordinal_input;
%let dm_num_ordinal_input = 0;
%macro dm_class_input;
   'Sex'n
%mend dm_class_input;
%global dm_num_class_input;
%let dm_num_class_input = 1 ;
%macro dm_segment;
%mend dm_segment;
%global dm_num_segment;
%let dm_num_segment = 0;
%macro dm_id;
   'journalid_lopnr'n 'taskid_lopnr'n
```

```
%mend dm_id;
%global dm_num_id;
%let dm_num_id = 2 ;
%macro dm_text;
%mend dm_text;
%global dm_num_text;
%let dm_num_text = 0;
%macro dm_strat_vars;
   'REDORANGE_YELLOWGREEN'n
%mend dm_strat_vars;
%global dm_num_strat_vars;
%let dm_num_strat_vars = 1 ;
*------------------------------------------------------------
*;
  * Component Code;
*------------------------------------------------------------
*;
proc gradboost
data=&dm_datalib..'DM_DIEUOP2FM88NAZUW3WHKOA7PF'n(&dm_data_cas
lib)
     earlystop(tolerance=0 stagnation=5 minimum=NO metric=MCR)
     binmethod=QUANTILE
     maxbranch=2
     assignmissing=USEINSEARCH minuseinsearch=1
     minleafsize=5
     seed=12345
     printtarget
  ;
  partition rolevar='_Partition_'n (TRAIN='Training'
VALIDATE='Validation' TEST='Test');
  autotune useparameters=CUSTOM tuningparameters=(
     lasso(LB=0 UB=10 INIT=0)
     learningrate(LB=0.01 UB=1 INIT=0.1)
     ntrees(LB=20 UB=150 INIT=100)
     ridge(LB=0 UB=10 INIT=1)
     samplingrate(LB=0.1 UB=1 INIT=0.5)
     maxdepth(LB=1 UB=6 INIT=4)
     numbin(LB=20 UB=100 INIT=50)
     vars_to_try(LB=1 UB=6 INIT=6)
     )
     searchmethod=GA objective=KS maxtime=3600
     maxevals=50 maxiters=5 popsize=10
     targetevent='1'
  ;
  target 'REDORANGE_YELLOWGREEN'n / level=nominal;
  input %dm_interval_input / level=interval;
  input %dm_binary_input %dm_nominal_input %dm_ordinal_input
%dm_unary_input  / level=nominal;
  ods output
     VariableImportance   = &dm_lib..varimportance
     Fitstatistics        = &dm_data_outfit
```

```
      PredProbName = &dm_lib..PredProbName
      PredIntoName = &dm_lib..PredIntoName
      TunerResults = &dm_lib..tuneresults
      BestConfiguration = &dm_lib..tunebest(drop=name)
   ;
   id 'journalid_lopnr'n 'taskid_lopnr'n;
   savestate
rstore=&dm_datalib.._B7C04JZNYHPNOS5YX3FFLGFR3_ast;
run;
```

C. Gradient Boosting Assessment

Root Average	Misclassification at Cutoff	Sum of Frequencies	Gini Coefficie	Gamma	Formatted Partition	Partition	Data Role	Misclassification Rate	ASE	Multi-Class Log Loss	Misclassification Rate	KS (Youden)	ROC Separation	Divisor for ASE	Tau	KS Cutoff	Area Under ROC
0.48	0.37	13239.00	0.32	0.35	Test	Test	TEST	0.42	0.23	0.59	0.37	0.24	0.20	13239.00	0.16	0.45	0.66
0.48	0.37	79437.00	0.34	0.37	Training	Training	TRAIN	0.42	0.23	0.58	0.36	0.26	0.20	79437.00	0.17	0.45	0.67
0.48	0.37	39719.00	0.33	0.37	Validation	Validation	VALIDATE	0.42	0.23	0.59	0.37	0.25	0.20	39719.00	0.16	0.45	0.67

View chart: Percentage Plot ▼

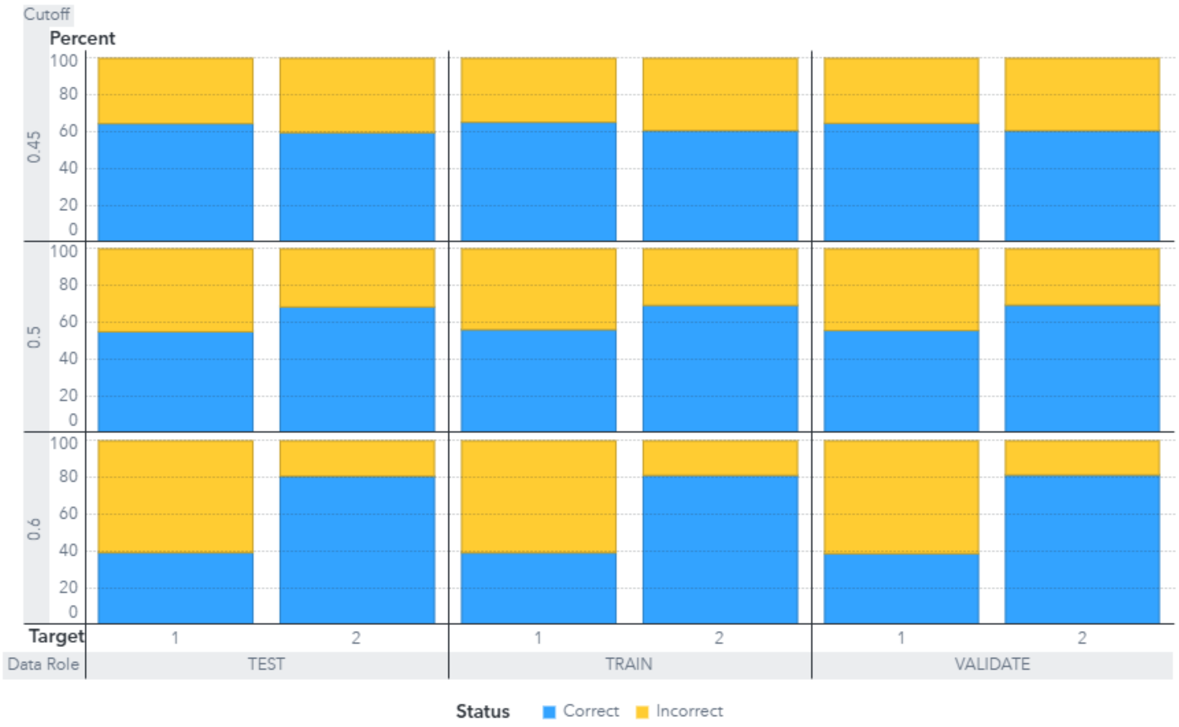

D. Variable importance

Model Variable Importance

Variable Label	Role	Variable Name	Training Importance	Importance Standard D...	Relative Importance
	INPUT	Age	22.6630	170.6281	1
	INPUT	Responsetime	14.6565	107.7238	0.6467
	INPUT	AvgResponsetimeH	3.6304	18.6278	0.1602
Sex	INPUT	Sex	1.5511	26.5285	0.0684
	INPUT	Hour	0.9113	2.8507	0.0402
	INPUT	Month	0.3971	2.4179	0.0175
